# Supplementary material for: Histone Recognition and Large-Scale Structural Analysis of the Human Bromodomain Family
Source: Cell. 2012 Mar 30;149(1):214–31. doi: 10.1016/j.cell.2012.02.013 (PMC3326523; doi:10.1016/j.cell.2012.02.013)
Supplement: Document S1. Tables S3, S4, and S6–S9 [file mmc4.pdf]

Supplemental Tables

Table S3. Available Bromodomain Structures, Related to Experimental Procedures

Protein	PDB Code	Resolution [Å]	Reference
ASH1L	3MQM	2.54	this study
ATAD2	3DAI	1.95	this study
BAZ2B	3G0L	2.03	this study
BPTF	2F6N, 3UV2	1.45, 1.58	(Li et al., 2006), this study
BRD1	3RCW	2.21	this study
BRD2(1)	2DVS	2.04	(Umehara et al., 2010)
BRD2(2)	2G4A	NMR	unpublished
BRD3(1)	2NXB	1.40	this study
BRD3(2)	2OO1	1.70	this study
BRD4(1)	2OSS	1.35	this study
BRD4(2)	2OUO	1.89	this study
BRD7	2I7K	NMR	(Sun et al., 2007)
BRD9	3HME	2.23	this study
BRDT(1)	2RFJ	2.05	this study
CECR2	3NXB	1.83	this study
EP300	3I3J	2.33	this study
CREBBP	3DWY	1.98	this study
GCN5L2	3D7C	2.06	this study
KIAA1240	3LXJ	2.33	this study
MLL	3LQH	1.72	(Wang et al., 2010)
PB1(1)	3IU5	1.63	this study
PB1(2)	3HMF	1.63	this study
PB1(3)	3K2J	2.20	this study
PB1(4)	3TLP	2.13	this study
PB1(5)	3G0J	1.78	this study
PB1(6)	3IU6	1.79	this study
PCAF	3GG3	2.25	this study
PHIP(2)	3MB3	2.25	this study
SMARCA2	2DAT	NMR	unpublished
SMARCA4	2GRC, 3UVD	1.50	(Singh et al., 2007), this study
TAF1(2)	3AAD, 3UV4	3.30, 1.89	(Akai et al., 2010), this study
TAF1(1/2)	1EQF, 3UV5	2.10, 2.03	(Jacobson et al., 2000), this study
TAF1L(2)	3HMH	2.05	this study
TIF1	3O33	2.00	(Tsai et al., 2010)
TRIM28	2RO1	NMR	(Zeng et al., 2008)
TRIM33	3U5M	3.1	(Xi et al., 2011)
WDR9(2)	3Q2E	1.74	this study

*Only the first structure deposited into the PDB has been included in the table. NMR structures are also available for several bromodomains. However, due to space limitations they have not been included unless no crystal structure is available for the target.

Table S4. Available Structures of BRD Peptide Complexes, Related to Experimental Procedures

Protein	PDB Code	Reso. [Å]	Histone Mark	Peptide/ Ligand	Reference
BPTF	2RI7	1.45	H3K4	H3(1-9)$\mathbf{K4_{me2}}$	(Li et al., 2006)
BPTF	2F6J	2.00	H3K4	H3(1-15)$\mathbf{K4_{me3}}$	(Li et al., 2006)
BPTF	2FSA	1.90	H4K4	H3(1-15)$\mathbf{K4_{me2}}$	(Li et al., 2006)
BPTF	3QZV	2.00	H4K12	H4(7-17)$\mathbf{K12_{ac}}$	(Ruthenburg et al., 2011)
BPTF	3QZS	1.80	H4K16	H4(12-21)$\mathbf{K16_{ac}}$	(Ruthenburg et al., 2011)
BPTF	3QZT	1.50	H4K20	H4(16-25)$\mathbf{K20_{ac}}$	(Ruthenburg et al., 2011)
BRD2(1)	2DVQ	2.04	H4K12	SGRGKGGKGLG$\mathbf{K_{ac}}$GGA	(Umehara et al., 2010)
BRD2(1)	2DVR	2.30	H4K12	GGKGLG$\mathbf{K_{ac}}$GGA	(Umehara et al., 2010)
BRD2(2)	2E3K	2.30	H4K5/K12	SGRG$\mathbf{K_{ac}}$GGKGLG$\mathbf{K_{ac}}$GGA	unpublished
BRD4(1)	3MUK	1.75	H3K23prop	AT$\mathbf{K_{prop}}$AARK	(Vollmuth and Geyer, 2010)
BRD4(1)	3MUL	1.65	H3K14buty	GK_{but}	(Vollmuth and Geyer, 2010)
BRD4(1)	3UVW	1.49	H4K5K8	SGRG$\mathbf{K_{ac}}$GG$\mathbf{K_{ac}}$GLGY	this study
BRD4(1)	3UVX	1.91	H4K12K16	G$\mathbf{K_{ac}}$GGA$\mathbf{K_{ac}}$RHRKV	this study
BRD4(1)	3UVY	2.02	H4K16K20	A$\mathbf{K_{ac}}$RHR$\mathbf{K_{ac}}$VLRDN	this study
BRD4(1)	3UW9	2.37	H4K8K12	G$\mathbf{K_{ac}}$GLG$\mathbf{K_{ac}}$GGAKR	this study
CREBBP	1JSP	NMR	p53K382	SHLKSKKGQSTSRHK$\mathbf{K_{ac}}$LMFK	(Mujtaba et al., 2004)
MLL	3LQI	1.92	H3K4	H3(1-9)$\mathbf{K4_{me2}}$	(Wang et al., 2010)
MLL	3LQJ	1.90	H3K4	H3(1-9)$\mathbf{K4_{me3}}$	(Wang et al., 2010)
PB1(2)	2KTB	NMR	H3K14	ARTKQTARKSTGG$\mathbf{K_{ac}}$APRKQL	(Charlop-Powers et al., 2010)
PCAF	2RNW	NMR	H3K9	ARTKQTAR$\mathbf{K_{ac}}$STGGKA	(Zeng et al., 2008)
PCAF	2RNX	NMR	H3K36	STGGV$\mathbf{K_{ac}}$KPHRYKC	(Zeng et al., 2008)
PCAF	2RNY	NMR	H4K20	GGAKRHR$\mathbf{K_{ac}}$VLRDNIQ	(Zeng et al., 2008)
PCAF	1JM4	NMR	HIV/Tat	SYGR$\mathbf{K_{ac}}$KRRQR	(Mujtaba et al., 2002)
TIF1α	3O34	1.90	H3K23	H3(13-32)$\mathbf{K23_{ac}}$	(Tsai et al., 2010)
TIF1α	3O35	1.76	H3K27	H3(23-31)$\mathbf{K27_{ac}}$	(Tsai et al., 2010)
TIF1α	3O36	1.70	H4K16	H4(14-19)$\mathbf{K16_{ac}}$	(Tsai et al., 2010)
TIF1α	3O37	2.00	H3K4	H3(1-10)$\mathbf{K4_{ac}}$	(Tsai et al., 2010)
TRIM33	3U5N	1.95	H3K14	H3(1-20)$K9_{me3}$$\mathbf{K14_{ac}}$	(Xi et al., 2011)
TRIM33	3U5O	2.70	H3K14K18	H3(1-22)$K9_{me3}$$\mathbf{K14_{ac}K18_{ac}}$	(Xi et al., 2011)
TRIM33	3U5P	2.80	H3K14K18K23	H3(1-28)$K9_{me3}$$\mathbf{K14_{ac}K18_{ac}K23_{ac}}$	(Xi et al., 2011)

Table S6. BRD Histone Interactions, Related to Results

Acetyl-lysine specific histone interactions identified in the array of peptides with singly acetylated lysine residues. Strong specific spots are shown in bold and weak intensity spots are shown in smaller fond size. Interactions confirmed by ITC are underlined, published quantified interactions are marked by "a" and published but not quantified ones are marked by "b". Single acetylation marks identified in the H3 microSPOT array are marked by "c".

	**Histone**				
**BRD**	**H1-4**	**H2A**	**H2B**	**H3**	**H4**
ASH1L	**K74**	**K36**	K30, K34, K33, K36, **K85**, K120	K37, **K56**, K79, K122	**K59**, **K79**
ATAD2	K74	**K36**	K85	K56	
BAZ2B	K89, K98	K36, K74, K129	K5, K11, K12, K34, K85, K108, K116	**K14**, K36, K37, K79, K115, K122	K31, K77, K79
BPTF	K25, K31, K33, K62, K80, K84, K105, K185,		K11, K12, K30, K34, K43	K18, K23, K27	K5, K8, K20,
BRD2(1)	**K74**, K80, K84, K89	K95	K5, K34	K37, K56, K115	K5[b], K44, K79
BRD3(1)		K127	K43, K120	K122, K27[c], K64[c]	K79
BRD3(2)		**K36**	K85		
BRD4(1)	K31, K33, K62, K63, K121[c]	K74, **K75**, K99,	K24, K116, K120, K125,	K4, K5, K18, K23, K27, **K36**, K37	K5, K20
BRD4(2)	K84, K194	**K5**, K9, K15, **K36**, K74, K75	**K43**, K46,	K14, **K18**, **K36**, **K37**, **K56**, K64, K115, K122	**K5**[a], **K20**, **K44**, K77, K79, K91
BRDT(1)	K33, K74, K80, K84, K89	K15, **K36**, K74, **K75**, K95, K99, K125, K129	K12, K30, K34, K116	K4, K9, K18, K23, K27, K36, K37, K56, K115, K122	K5, K8, K20, K37, K44, K91
BRD9				K18[c], K14[c], K27[c], K36[c], K56[c], K115[c]	
BRPF1	K74	**K15**		**K56**, K115, K122	**K44**, **K77, K79, K91**
CECR2		**K15, K36, K75**		K9[c]**,** K14[c]**, K18**, K27[c], K36, K56, K64[c], K79[c], K115[c]	
CREBBP	K74	**K15**		K34, K43, K36[a], K37, **K56**, K64, K122	K44, K59, K77, K79, K91
EP300	K31, K33, K51, K63, **K74,** K116	**K5**, **K15**, **K36**, K74, K75,	K23, K34, **K43**, **K46,** K108, K116, K116, K120, K125	K4, K14, K18, K23, K36, K37, **K56**, K64, **K79**, **K115, K122,**	**K5**, K8, K12, **K44**, K77, K79, K91
GCN5L2	K31, K33, K138, K139, K174, K175, K189, K191, K194	K5, K9, K125, K127, K129		K4, K9, K36, K37, K56[c]	K20

	K196, K199, K211, K212, K216, K218				
KIAA1240	**K74**	K5, K15, **K36**	**K43**, K46,	K9, **K56**, K115, K122	**K5**, **K59**, K77, K79
LOC93349	K22, K128, K135, K136, K138, K139, K158, K159, K170, K173, K174, K176, K182, K185, K211, K212, K216, K217, K218	K119, K125, K127, K129	K27, K30, K34		**K5**, **K8**, K12, K16, K20
MLL		K15			
PB1(1)	K33, K80, K84, K89	K15		K56, K64, K122	K44, K91
PB1(2)		K36	K85	K14[a]	
PB1(3)	K62, K74, K80, K84	K15	K23,	K18[a], K36[a], K56, K64[c]	
PB1(4)				K37[a,c], K122[c]	
PB1(5)			**K85**		
PB1(6)	K84		K43	K56, K115[c]	
PCAF	K25, K31, K33, K105, K108, K109, K151, K152, K155, K156, K158, K159	K99, K116, K119, K125, K127			K12, K16, K20[a], K59
PHIP(2)	K31, K33, **K62**, K80, K84, K89			K9, K18, K36, K37	**K91**
SMARCA2	K33, K74, K170, K182	K15, K95, K107	**K5, K34**	**K14**[b], K18, K23, K27[c], K36, **K37**, K56	K5, K8[b],
SMARCA4	K80, K84, K105	K75,	K20, K23, K24	K18, K23, K27, K36	
SP140				K14[c], K23[c], K36[c]	
TAF1(1)	K74	K15, **K36**		K56	
TAF1L(1)		K15, K36	K85		
TAF1(2)	K31, K62, K105	**K15**, K75	K116, K120	K18, K56, K115, K64[c]	**K44**, **K77**, K79
TAF1L(2)	**K31**, K33, K84, K105, K118, K147, K148	K74, K75	K11, K12, K20, K23, K24, K116, K120, K125	K4, K9, K14, K23, K27,	K5, K8, **K77**, K79, **K91**
TIF1	K31, K33, K62, K63, K84, K116, K147, K148, K182	**K15**, **K36**, K74, K75, K99,	K12, K20	K4, K9[a], K14[a], K18, K23[a], K27, K36, K37, **K56**, K64, K115	K5, **K44**, K31, K77, K79, **K91**
TRIM28				K37[c]	

Table S7. ITC Data of Monoacetylated Histone Peptides, Related to Results

Protein	Histone Mark	Histone Peptide Sequence	[Protein] (μM)	[Peptide] (mM)	N			K_D (μM)			ΔH (kcal/mol)	TΔS (kcal/mol)	ΔG (kcal/mol)
ATAD2	H3K9	KQTAR**K**$_{ac}$STGGK**Y**	55	1.58		NB							
BAZ2B	H3K14	KSTGG**K**$_{ac}$APRKQ**Y**	50	1.55	1.00	±	0.007	7.6	±	0.3	-8.91	-2.27	-6.64
BRD2(1)	H4K5	SGRG**K**$_{ac}$GGKGL**Y**	53	1.52	1.01	±	0.056	130.2	±	4.4	-9.78	-4.75	-5.03
BRD3(1)	H4K8	RGKGG**K**$_{ac}$GLGKG**Y**	56	1.54	1.00	±	0.000	211.0	±	5.2	-3.07	1.69	-4.76
BRD3(2)	H3K18	GKAPR**K**$_{ac}$QLATK**Y**	51	1.55	1.06	±	0.068	67.1	±	4.5	-4.15	1.26	-5.41
	H4K12	GKGLG**K**$_{ac}$GGAKR**Y**	57	1.50	1.00	±	0.096	149.5	±	8.0	-4.80	0.15	-4.96
	H4K20	AKRHR**K**$_{ac}$VLRDN**Y**	58	1.47	1.00	±	0.005	10.5	±	0.2	-4.70	1.75	-6.45
BRD4(1)	H3K9	KQTAR**K**$_{ac}$STGGK**Y**	55	1.48	1.01	±	0.467	301.2	±	40.9	-1.22	3.34	-4.56
	H4K8	RGKGG**K**$_{ac}$GLGKG**Y**	60	1.58	1.03	±	0.106	84.7	±	8.2	-2.00	3.25	-5.26
BRD4(2)	H3K14	KSTGG**K**$_{ac}$APRKQ**Y**	54	1.48	1.03	±	0.383	260.4	±	32.2	-6.80	-2.15	-4.64
	H4K5	SGRG**K**$_{ac}$GGKGL**Y**	51	1.58	1.00	±	0.000	60.2	±	2.5	-4.66	0.80	-5.46
	H4K31	IQGIT**K**$_{ac}$PAIRR**Y**	55	1.57	1.01	±	0.122	170.4	±	10.1	-2.69	2.19	-4.88
BRDT(1)	H4K12	GKGLG**K**$_{ac}$GGAKR**Y**	57	1.58	0.96	±	0.180	149.9	±	14.6	-4.70	0.26	-4.95
CECR2	H3K9	KQTAR**K**$_{ac}$STGGK**Y**	59	1.51	1.00	±	0.011	189	±	11.6	-6.11	-1.29	-4.82
	H3K14	KSTGG**K**$_{ac}$APRKQ**Y**	56	1.50	1.00	±	0.005	43.9	±	0.8	-7.60	-1.95	-5.65
CREBBP	H2AK36	LLR**K**$_{ac}$GNYSERVGA	52	1.90		NB							
	H2BK85	AHYN**K**$_{ac}$RSTITSRE	53	1.90	0.98	±	0.059	66.2	±	3.8	-3.38	2.03	-5.41
	H3K14	KSTGG**K**$_{ac}$APRKQ**Y**	56	1.70	1.03	±	0.085	733.7	±	90.6	-6.05	-1.99	-4.06
	H3K27	TKAAR**K**$_{ac}$SAPAT**Y**	58	1.59		NB							
	H3K36	ATGGV**K**$_{ac}$KPHR**Y**	55	1.50	1.00	±	0.000	71.4	±	1.0	-5.57	-0.20	-5.37
	H3K37	ATGGVK**K**$_{ac}$PHRYRPG	53	1.30		NB							
	H3K56	GTVALREIRRYQ**K**$_{ac}$S	52	2.00	0.96	±	0.025	21.3	±	1.4	-12.02	-5.97	-6.30
	H3K56	IRRYQ**K**$_{ac}$STELLY	51	0.80	0.97	±	0.013	13.8	±	0.4	-7.91	-1.61	-5.80
	H4K12	GKGLG**K**$_{ac}$GGAKR**Y**	59	1.51	1.00	±	0.026	48.1	±	1.9	-7.21	-1.61	-5.60
	H4K20	AKRHR**K**$_{ac}$VLRDN**Y**	52	1.50	1.14	±	0.027	41.5	±	1.8	-2.74	2.94	-5.69
	H4K44	GGV**K**$_{ac}$RISGLI	50	1.60	1.06	±	0.010	82.0	±	3.8	-3.36	1.94	-5.29
	H4K44	RRGGV**K**$_{ac}$RISGL**Y**	59	1.50	1.00	±	0.099	25.3	±	0.6	-4.20	1.76	-5.96

EP300	H3K27	TKAAR**K**$_{ac}$SAPAT**Y**	57	1.47		NB							
	H3K36	ATGGV**K**$_{ac}$KPHR**Y**	59	1.51	1.00	±	0.044	109.4	±	3.8	-5.66	-0.53	-5.13
	H3K56	GTVALREIRRYQ**K**$_{ac}$S	55	1.80	0.98	±	0.009	13.4	±	0.5	-12.22	-5.91	-6.31
	H4K12	GKGLG**K**$_{ac}$GGAKR**Y**	52	1.51	0.99	±	0.020	59.5	±	1.4	-8.06	-2.58	-5.48
	H4K20	AKRHR**K**$_{ac}$VLRDN**Y**	52	1.55	0.98	±	0.001	54.6	±	1.3	-4.50	1.03	-5.52
	H4K44	GGV**K**$_{ac}$RISGLI	55	2.20	0.97	±	0.088	131.9	±	6.3	-3.63	1.40	-5.03
FALZA	H4K5	SGRG**K**$_{ac}$GGKGL**Y**	53	1.6	1.00	±	0.170	175.4	±	13.3	-3.64	1.23	-4.86
	poly-R	RRRRR**K**$_{ac}$RRRRR**Y**	57	1.51	1.00	±	0.000	3.1	±	0.1	-10.09	-2.97	-7.12
GCN5L2	H4K8	SGRG**K**$_{ac}$GGKGL**Y**	51	1.58		NB							
KIAA1240	H4K5	RGKGG**K**$_{ac}$GLGKG**Y**	58	1.58	1.00	±	0.000	149.7	±	6.4	-2.31	2.64	-4.95
LOC93349	H3K9	KQTAR**K**$_{ac}$STGGK**Y**	53	1.48		NB							
PB1(1)	H3K14	KSTGG**K**$_{ac}$APRKQ**Y**	54	1.54		NB							
	H4K20	AKRHR**K**$_{ac}$VLRDN**Y**	54	1.47		NB							
PB1(5)	H4K16	GKGGA**K**$_{ac}$RHRKV**Y**	53	1.5		NB							
PCAF	H3K14	KSTGG**K**$_{ac}$APRKQ**Y**	58	1.51	1.01	±	0.162	188.3	±	14.6	-3.26	1.56	-4.83
	H4K16	GKGGA**K**$_{ac}$RHRKV**Y**	49	1.54	1.03	±	0.180	102.6	±	11.2	-1.69	3.48	-5.17
	H4K20	AKRHR**K**$_{ac}$VLRDN**Y**	55	1.47	0.97	±	0.140	90.9	±	9.6	-1.22	4.02	-5.23
SMARCA2	H3K14	KSTGG**K**$_{ac}$APRKQ**Y**	52	1.6	1.05	±	0.437	286.5	±	35.2	-2.96	1.63	-4.59
	H4K16	GKGGA**K**$_{ac}$RHRKV**Y**	53	1.6	1.00	±	0.001	86.2	±	3.0	-1.77	3.48	-5.25
TIF1	H4K16	GKGGA**K**$_{ac}$RHRKV**Y**	59	1.59	1.00	±	0.023	92.6	±	1.9	-8.18	-2.94	-5.24
TAF1(1)	H3K9	KQTAR**K**$_{ac}$STGGK**Y**	51	1.5		NB							
	H3K14	KSTGG**K**$_{ac}$APRKQ**Y**	50	1.64		NB							
TAF1(2)	H3K9	KQTAR**K**$_{ac}$STGGK**Y**	52	1.5		NB							
	H3K14	KSTGG**K**$_{ac}$APRKQ**Y**	54	1.52	1.03	±	0.231	305.8	±	21.4	-0.95	3.59	-4.55
TAF1(1/2)	H3K9	KQTAR**K**$_{ac}$STGGK**Y**	47	1.5		NB							
	H3K14	KSTGG**K**$_{ac}$APRKQ**Y**	50	1.5	1.01	±	0.083	178.9	±	6.4	-0.79	4.08	-4.86

Table S8. ITC Data of Polyacetylated Histone Peptides, Related to Results

Protein	Acetylated Histone Marks	Histone Peptide Sequence	Protein (μM)	Pep. (mM)	N[&]			K_D (μM)			ΔH (kcal/mol)	TΔS (kcal/mol)	ΔG (kcal/mol)
	H4K5/K8	SGRG$\mathbf{K}_{ac}$GG$\mathbf{K}_{ac}$GLGY	55.70	1.84	0.99	±	0.006	13.5	±	0.3	-16.44	-10.13	-6.31
	H4K8/12	G$\mathbf{K}_{ac}$GLG$\mathbf{K}_{ac}$GGAKR	53.00	1.50	1.00	±	0.050	65.8	±	3.5	-4.60	0.82	-5.42
BRD2(1)	H4K12/16	G$\mathbf{K}_{ac}$GGA$\mathbf{K}_{ac}$RHRKV	47.60	2.29	1.00	±	0.019	29.4	±	1.0	-5.77	0.10	-5.87
	H4K12/16/20	G$\mathbf{K}_{ac}$GGA$\mathbf{K}_{ac}$RHR$\mathbf{K}_{ac}$V	47.00	1.26	0.50	±	0.009	27.9	±	0.7	-18.81	-12.90	-5.91
	H4K5/8/12/16	YSGRG$\mathbf{K}_{ac}$GG$\mathbf{K}_{ac}$GLG$\mathbf{K}_{ac}$GGA$\mathbf{K}_{ac}$RHRK	50.00	1.45	0.53	±	0.003	3.7	±	0.1	-30.55	-23.52	-7.03
	H4K5/K8	SGRG$\mathbf{K}_{ac}$GG$\mathbf{K}_{ac}$GLGY	55.00	1.38	0.96	±	0.015	32.8	±	1.6	-3.57	2.24	-5.81
	H4K8/12	G$\mathbf{K}_{ac}$GLG$\mathbf{K}_{ac}$GGAKR	49.70	1.50	0.51	±	0.023	55.9	±	1.5	-12.51	-6.99	-5.52
BRD2(2)	H4K12/16	G$\mathbf{K}_{ac}$GGA$\mathbf{K}_{ac}$RHRKV	50.70	1.50	1.00	±	0.017	27.2	±	1.0	-3.51	2.40	-5.91
	H4K12/16/20	G$\mathbf{K}_{ac}$GGA$\mathbf{K}_{ac}$RHR$\mathbf{K}_{ac}$V	51.73	1.26	0.50	±	0.024	41.0	±	1.5	-11.05	-5.38	-5.67
	H4K5/8/12/16	YSGRG$\mathbf{K}_{ac}$GG$\mathbf{K}_{ac}$GLG$\mathbf{K}_{ac}$GGA$\mathbf{K}_{ac}$RHRK	50.70	1.60	0.50	±	0.033	34.7	±	2.0	-7.02	-1.24	-5.78
BRD3(1)	H4K12/16/20	G$\mathbf{K}_{ac}$GGA$\mathbf{K}_{ac}$RHR$\mathbf{K}_{ac}$V	54.00	1.50	0.51	±	0.014	31.4	±	0.9	-19.09	-13.24	-5.85
BRD3(2)	H4K12/16/20	G$\mathbf{K}_{ac}$GGA$\mathbf{K}_{ac}$RHR$\mathbf{K}_{ac}$V	51.43	1.50	0.50	±	0.027	61.3	±	1.9	-12.42	-6.96	-5.46
	H4K5/K8	SGRG$\mathbf{K}_{ac}$GG$\mathbf{K}_{ac}$GLGY	56.35	1.94	1.00	±	0.003	6.8	±	0.1	-15.75	-9.06	-6.69
	H4K8/12	G$\mathbf{K}_{ac}$GLG$\mathbf{K}_{ac}$GGAKR	58.00	1.50	0.50	±	0.012	27.4	±	0.9	-10.33	-4.41	-5.92
BRD4(1)	H4K12/16	G$\mathbf{K}_{ac}$GGA$\mathbf{K}_{ac}$RHRKV	57.70	2.35	1.02	±	0.013	46.1	±	0.9	-7.73	-2.11	-5.62
	H4K12/16/20	G$\mathbf{K}_{ac}$GGA$\mathbf{K}_{ac}$RHR$\mathbf{K}_{ac}$V	56.00	1.26	0.50	±	0.012	20.4	±	0.8	-15.94	-9.85	-6.09
	H4K5/8/12/16	YSGRG$\mathbf{K}_{ac}$GG$\mathbf{K}_{ac}$GLG$\mathbf{K}_{ac}$GGA$\mathbf{K}_{ac}$RHRK	54.00	1.50	0.57	±	0.005	2.8	±	0.2	-28.59	-21.39	-7.20
	H4K5/K8	SGRG$\mathbf{K}_{ac}$GG$\mathbf{K}_{ac}$GLGY	58.00	1.75	0.50	±	0.031	63.3		2.3	-11.64	-6.20	-5.44
BRD4(2)	H4K8/12	G$\mathbf{K}_{ac}$GLG$\mathbf{K}_{ac}$GGAKR	50.00	1.50	0.50	±	0.013	20.4	±	0.8	-13.59	-7.50	-6.09
	H4K12/16	G$\mathbf{K}_{ac}$GGA$\mathbf{K}_{ac}$RHRKV	50.76	2.27	1.01	±	0.018	49.3	±	1.1	-7.25	-1.66	-5.58
	H4K12/16/20	G$\mathbf{K}_{ac}$GGA$\mathbf{K}_{ac}$RHR$\mathbf{K}_{ac}$V	54.00	1.50	0.50	±	0.012	22.7	±	0.8	-12.71	-6.68	-6.03

	H4K5/8/12/16	YSGRG**K**$_{ac}$GG**K**$_{ac}$GLG**K**$_{ac}$GGA**K**$_{ac}$RHRK	51.30	1.60	0.25	±	0.018	26.6	±	1.1	-23.02	-17.06	-5.96
BRD4(1/2)	H4K5/8/12/16	YSGRG**K**$_{ac}$GG**K**$_{ac}$GLG**K**$_{ac}$GGA**K**$_{ac}$RHRK	52.00	1.60	0.70	±	0.007	2.7	±	0.2	-21.02	-13.81	-7.21
BRDT(1)	H4K5/8/12/16	YSGRG**K**$_{ac}$GG**K**$_{ac}$GLG**K**$_{ac}$GGA**K**$_{ac}$RHRK	54.00	1.50	2.04	±	0.078	23.0	±	0.5	-7.70	-1.69	-6.01
CREBBP	H3S10K14K18	K**pS**TGG**K**$_{ac}$APR**K**$_{ac}$Q**Y**	49.00	1.60	1.00	±	0.093	131	±	6.5	-4.73	0.30	-5.03
TAF1(1/2)	H3K9/K14	KQTAR**K**$_{ac}$STGG**K**$_{ac}$APRKQ**Y**	56.00	1.49	1.02	±	0.215	339	±	21	-2.33	2.16	-4.49
	H3K9/S10/K14	KQTAR**K**$_{ac}$**pS**TGG**K**$_{ac}$APRKQ**Y**	58.00	1.58	1.05	±	0.099	150	±	8.7	-0.77	4.16	-4.93
CECR2[≠]	H3K9K14	KQTAR**K**$_{ac}$STGG**K**$_{ac}$APRKQ**Y**	55.00	1.55	1.00	±	0.000	57.8	±	1.9	-10.70	-4.59	-5.49
						±	0.000	164.5	±	5.5	-10.07	-5.15	-4.90
	H3K9/S10/K14	KQTAR**K**$_{ac}$**pS**TGG**K**$_{ac}$APRKQ**Y**	55.00	1.49	1.00	±	0.000	51.0	±	1.8	-6.42	-0.86	-5.56
						±	0.000	609.8	±	48.0	-6.95	-2.78	-4.17

N[&]: For binding stoichiometries of 0.5 or 0.25, two or more bromodomains interact with one peptide. The derived binding constants have to be therefore considered as apparent binding constants since the different binding events could not be deconvoluted.

[≠]: Deconvoluted binding constants using a sequential binding model (Klosi et al., 2007).

Table S9. Effect of Linker Sequence to Peptide Binding on the Bromodomains of BRD4 Determined by ITC, Related to Results

Protein	Peptide	Histone Peptide Sequence	Protein (μM)	Pep. (mM)	N[&]	K_D (μM)	ΔH (kcal/mol)	TΔS (kcal/mol)	ΔG (kcal/mol)
BRD4(1)	**Linker = 3 Gly**[#]	Biotin-SGRG$\mathbf{K}_{ac}$GGG$\mathbf{K}_{ac}$GLG**Y**	59.0	1.49	1.00 ± 0.05	83.3 ± 3.5	-8.78	-3.48	-5.30
	**Linker = 2 Gly**[#]	Biotin-SGRG$\mathbf{K}_{ac}$GG$\mathbf{K}_{ac}$GLG**Y**	44.5	2.10	0.98 ± 0.01	7.0 ± 0.3	-15.62	-8.94	-6.68
	**Linker = 1 Gly**[#]	Biotin-SGRG$\mathbf{K}_{ac}$G$\mathbf{K}_{ac}$GLG**Y**	59.0	1.63	0.99 ± 0.06	108.5 ± 4.5	-7.73	-2.59	-5.14
	**Linker = 0 Gly**[#]	Biotin-SGRG$\mathbf{K}_{ac}\mathbf{K}_{ac}$GLG**Y**	55.6	1.59	1.02 ± 0.35	285.7 ± 28.2	-9.46	-4.87	-4.59
	**H4K5**	Biotin-SGRG$\mathbf{K}_{ac}$GGKGLG**Y**	55.6	1.71	1.06 ± 0.25	215.1 ± 18.5	-8.70	-3.93	-4.77
	**H4K8**	Biotin-SGRGKGG$\mathbf{K}_{ac}$GLG**Y**	55.6	1.80	very weak interaction (ΔH < 1 Kcal)				
	**H4K5/16**	Biotin-SGRG$\mathbf{K}_{ac}$GGKGLGKGGA$\mathbf{K}_{ac}$RHR**Y**	55.6	1.61	1.02 ± 0.07	92.6 ± 4.3	-6.50	-1.27	-5.23
**ΔBRD4(1)^{N140A}**	**H4K5/8**	SGRG$\mathbf{K}_{ac}$GG$\mathbf{K}_{ac}$GLG**Y**	60.0	2.10	No binding				
BRD4(2)	**Linker = 3 Gly**[#]	Biotin-SGRG$\mathbf{K}_{ac}$GGG$\mathbf{K}_{ac}$GLG**Y**	46.1	1.49	1.01 ± 0.10	135.3 ± 5.6	-8.48	-3.45	-5.03
	**Linker = 2 Gly**[#]	Biotin-SGRG$\mathbf{K}_{ac}$GG$\mathbf{K}_{ac}$GLG**Y**	46.1	2.10	0.54 ± 0.03	36.5 ± 3.6	-5.92	-0.17	-5.75
	**Linker = 1 Gly**[#]	Biotin-SGRG$\mathbf{K}_{ac}$G$\mathbf{K}_{ac}$GLG**Y**	46.1	1.53	1.14 ± 0.04	44.4 ± 1.9	-5.18	0.46	-5.64
	**Linker = 0 Gly**[#]	Biotin-SGRG$\mathbf{K}_{ac}\mathbf{K}_{ac}$GLG**Y**	46.1	1.60	1.01 ± 0.07	68.0 ± 3.4	-6.67	-1.27	-5.40
	**H4K5**	Biotin-SGRG$\mathbf{K}_{ac}$GGKGLG**Y**	46.1	1.71	1.03 ± 0.15	155.5 ± 8.8	-5.90	-0.97	-4.93
	**H4K8**	Biotin-SGRGKGG$\mathbf{K}_{ac}$GLG**Y**	46.1	1.80	very weak interaction (ΔH < 1 Kcal)				
	**H4K5/16**	Biotin-SGRG$\mathbf{K}_{ac}$GGKGLGKGGA$\mathbf{K}_{ac}$RHR**Y**	46.1	1.61	1.00 ± 0.08	59.5 ± 3.7	-4.08	1.40	-5.47
**ΔBRD4(2)^{N433A}**	**H4K5/8**	SGRG$\mathbf{K}_{ac}$GG$\mathbf{K}_{ac}$GLG**Y**	58.5	2.10	No binding				

[#] The parent sequence used to probe the effect of the linker between K_{ac} marks is that of H4K5$_{ac}$K8$_{ac}$ (SGRG$\mathbf{K}_{ac}$GG$\mathbf{K}_{ac}$GLG)

N[&]: For binding stoichiometries of 0.5, two bromodomains interact with one peptide. The derived binding constants have to be therefore considered as apparent binding constants since the different binding events could not be deconvoluted.
