# Supplementary material for: Histone Recognition and Large-Scale Structural Analysis of the Human Bromodomain Family
Source: Cell. 2012 Mar 30;149(1):214–31. doi: 10.1016/j.cell.2012.02.013 (PMC3326523; doi:10.1016/j.cell.2012.02.013)
Supplement: Document S2. Article plus Supplemental Information [file mmc5.pdf]

Cell

Resource

Histone Recognition and Large-Scale Structural Analysis of the Human Bromodomain Family

Panagis Filippakopoulos,[1,*] Sarah Picaud,[1] Maria Mangos,[3] Tracy Keates,[1] Jean-Philippe Lambert,[5] Dalia Barsyte-Lovejoy,[3] Ildiko Felletar,[1] Rudolf Volkmer,[6] Susanne Müller,[1] Tony Pawson,[5,7] Anne-Claude Gingras,[5,7] Cheryl H. Arrowsmith,[3,4] and Stefan Knapp[1,2,8,*]
[1]Nuffield Department of Clinical Medicine, Structural Genomics Consortium
[2]Department of Oncology
University of Oxford, Old Road Campus Research Building, Roosevelt Drive, Oxford OX3 7LD, UK
[3]Structural Genomics Consortium
[4]Ontario Cancer Institute, Campbell Family Cancer Research Institute and Department of Medical Biophysics
University of Toronto, Toronto, Ontario M5G 1L7, Canada
[5]Centre for Systems Biology, Samuel Lunenfeld Research Institute, 600 University Avenue, Toronto, Ontario M5G 1X5, Canada
[6]Institut für Medizinische Immunologie, Charité-Universitätsmedizin Berlin, Hessische Str. 3-4, 10115 Berlin, Germany
[7]Department of Molecular Genetics, University of Toronto, 1 Kings College Circle, Toronto, Ontario M5S 1A8, Canada
[8]Department of Biochemistry and Molecular Biology, George Washington University, School of Medicine and Health Sciences, 2300 Eye Street, NW, Suite 530, Washington, DC, 20037, USA
*Correspondence: panagis.filippakopoulos@sgc.ox.ac.uk (P.F.), stefan.knapp@sgc.ox.ac.uk (S.K.)
DOI 10.1016/j.cell.2012.02.013

SUMMARY

Bromodomains (BRDs) are protein interaction modules that specifically recognize ε-N-lysine acetylation motifs, a key event in the reading process of epigenetic marks. The 61 BRDs in the human genome cluster into eight families based on structure/sequence similarity. Here, we present 29 high-resolution crystal structures, covering all BRD families. Comprehensive crossfamily structural analysis identifies conserved and family-specific structural features that are necessary for specific acetylation-dependent substrate recognition. Screening of more than 30 representative BRDs against systematic histone-peptide arrays identifies new BRD substrates and reveals a strong influence of flanking posttranslational modifications, such as acetylation and phosphorylation, suggesting that BRDs recognize combinations of marks rather than singly acetylated sequences. We further uncovered a structural mechanism for the simultaneous binding and recognition of diverse diacetyl-containing peptides by BRD4. These data provide a foundation for structure-based drug design of specific inhibitors for this emerging target family.

INTRODUCTION

ε-N-acetylation of lysine residues (K_{ac}) is one of the most frequently occurring posttranslational modifications (PTMs) in proteins (Choudhary et al., 2009). Acetylation has a profound effect on the physiochemical properties of modified lysine residues neutralizing the positive charge of the ε-amino group (Kouzarides, 2000). Lysine acetylation is abundant in large macromolecular complexes that function in chromatin remodeling, DNA damage, and cell-cycle control (Choudhary et al., 2009) and particularly in histones. Cellular acetylation levels are stringently controlled by two enzyme families: the histone acetyltransferases (HATs) and histone deacetylases (HDACs) (Shahbazian and Grunstein, 2007). Histone acetylation has been associated with transcriptional activation, but specific marks have also been linked to DNA repair (Kouzarides, 2007).

Bromodomains (BRDs) are protein interaction modules that exclusively recognize acetylation motifs. BRDs are evolutionarily conserved and present in diverse nuclear proteins comprising HATs (GCN5, PCAF), ATP-dependent chromatin-remodeling complexes (BAZ1B), helicases (SMARCA), methyltransferases (MLL, ASH1L), transcriptional coactivators (TRIM/TIF1, TAFs) transcriptional mediators (TAF1), nuclear-scaffolding proteins (PB1), and the BET family (Muller et al., 2011) (Figure 1A and Table 1). Despite large sequence variations, all BRD modules share a conserved fold that comprises a left-handed bundle of four α helices (α_Z, α_A, α_B, α_C), linked by loop regions of variable length (ZA and BC loops), which line the K_{ac} binding site and determine binding specificity. Cocrystal structures with peptides have demonstrated that K_{ac} is recognized by a central deep hydrophobic cavity, where it is anchored by a hydrogen bond to an asparagine residue present in most BRDs (Owen et al., 2000).

Dysfunction of BRD proteins has been linked to development of several diseases. For instance, recurrent t(15;19) chromosomal translocations that result in a fusion protein that comprises both BRD4 or BRD3 and the NUT (*nu*clear protein in *t*estis) lead to an aggressive form of human squamous

carcinoma (French, 2010a; French et al., 2001). Deregulation of transcription as a consequence of altered protein acetylation patterns is a hallmark of cancer, a mechanism that is currently targeted by HDAC inhibitors (Lane and Chabner, 2009). It is likely that selective inhibitors capable of targeting BRDs will find broad application in medicine and basic research as exemplified by the recent development of highly specific and potent acetyl-lysine competitive BET BRD inhibitors (Chung et al., 2011; Dawson et al., 2011; Delmore et al., 2011; Filippakopoulos et al., 2010; Mertz et al., 2011; Nicodeme et al., 2010).

To date, only a small number of lysine acetylation marks have been identified to specifically interact with individual BRDs, and the often weak affinities reported for BRD interactions with their potential target sites have been determined by a variety of different techniques making data comparison difficult (Muller et al., 2011). Reported affinities range from nano- to millimolar dissociation constant (K_D) values raising the issue of which affinity window is relevant for specific BRD-peptide interactions. For instance, the BRDs of BRD2 have been shown to bind histone 4 acetylated at lysine 12 ($H4K12_{ac}$) (Kanno et al., 2004), with K_D values that range from 360 μM for the diacetylated peptide $H4K5_{ac}K12_{ac}$ (Umehara et al., 2010) to 2.9 mM for the monoacetylated $H4K12_{ac}$ peptide (Huang et al., 2007). Closely spaced multiple K_{ac} sites have also been shown to significantly increase affinity of the histone H4 N terminus for BRDT by simultaneous binding to the same BRD (Morinière et al., 2009). Thus, the field would greatly benefit from a more systematic analysis of BRD structure and peptide binding properties in order to better understand acetylation-mediated signaling as interpreted through BRDs.

Here, we present a comprehensive structural characterization of the human BRD family together with identified K_{ac}-specific interaction sites of these essential protein recognition modules with their target sites in histones. Using available sequence databases, we identified 61 BRDs in the human proteome that are present in 46 diverse proteins. High-throughput cloning led to the establishment of 171 expression systems that yielded functional recombinant proteins. Using these reagents, we crystallized and determined the structures of 29 BRDs, including 25 structures that had not been previously published. We performed a SPOT blot analysis that covered all possible K_{ac} sites of human histones (Nady et al., 2008) for 43 members of the BRD family. We identified 485 linear K_{ac}-dependent BRD-binding motifs, and determined accurate binding affinities in solution for 81 known cellular histone marks by isothermal titration calorimetry (ITC). Furthermore, we found that BRD peptide recognition is dependent on patterns of multiple modifications rather than on a single acetylation site. This study provides a comprehensive structural comparison of this protein family interpreted in the context of a large array of histone interaction data, establishing a powerful resource for future functional studies of this family of epigenetic reader domains.

RESULTS

The Human BRD Family

Analysis of sequence databases (NCBI, UniProt, PFAM) identified 46 diverse human proteins that contain a total of 61 diverse BRDs. BRD-containing proteins are large multidomain proteins associated with chromatin remodeling, transcriptional control, methyl or acetyltransferase activity, or helicases (Figure 1A and Table 1). The domain organization in BRD-containing proteins is evolutionarily highly conserved, and the BRD motif is often flanked by other epigenetic reader domains. Most frequently observed combinations include the presence of plant homeodomains (PHDs) N terminal to the BRD, multiple BRDs, as well as various other domains that generally mediate protein interactions such as bromo-adjacent homology (BAH) domains (Goodwin and Nicolas, 2001).

Phylogenetic analysis of the BRD family outside the two central core helices was complicated by the low-sequence homology and nonconserved insertions in BRD loop regions. We therefore used three-dimensional structure-based alignments including available NMR models together with secondary structure prediction (Jones, 1999) and manual curation of the aligned sequences to establish an alignment of all human BRDs (Figure S1). The derived phylogram clustered into eight major BRD families designated by Roman numerals (I–VIII) (Figure 1B). Key references for all BRD-containing proteins are included in Table 1.

Multiple protein interaction modules can be tightly linked to form a single stable interaction domain, or they can be connected by flexible linker sequences allowing conformational adaptation to diverse sequences motifs. An example of a tightly linked dual-domain reader is the PHD-BRD of TRIM24, in which both domains interact through a large interface that orients both peptide binding cavities to the same side of the protein (Tsai et al., 2010). In contrast, the two BRDs in TAF1 are free to orient independently as shown by the different domain orientations in the dual-domain structure determined here and a previously published model (Jacobson et al., 2000) (Figure 1C). The frequent combination of multiple interaction modules in the same protein suggests that the epigenetic reading process involves concomitant recognition of several PTMs.

Structural Analysis

In order to establish a platform of recombinant BRDs for functional and structural studies, we subcloned all human BRDs into bacterial expression systems in frame with a cleavable N- (or C-) terminal His_6 tag. A total of 1,031 constructs resulted in the identification of 171 expression systems covering 44 BRDs that yielded stable and soluble proteins. Details of the cloned constructs are summarized in Table S1, and descriptions of one representative expression system per BRD are summarized in Table S2. The expressed proteins provide an excellent coverage of representative BRDs of all eight families.

A total of 133 recombinant BRD constructs covering 44 unique BRDs were expressed at levels sufficient for structural studies, resulting in the determination of a total of 33 crystal structures of apo-BRDs (Table S3), or BRDs in complex with acetylated peptides (Table S4). Together with previously published structural information, each BRD family is represented by at least one structural model, and families I, II, and VIII are either completely or nearly completely covered (Figure 1B). All structures presented here were refined at high resolution. A summary of the crystallization conditions, data collection, and refinement statistics is compiled in Table S5.

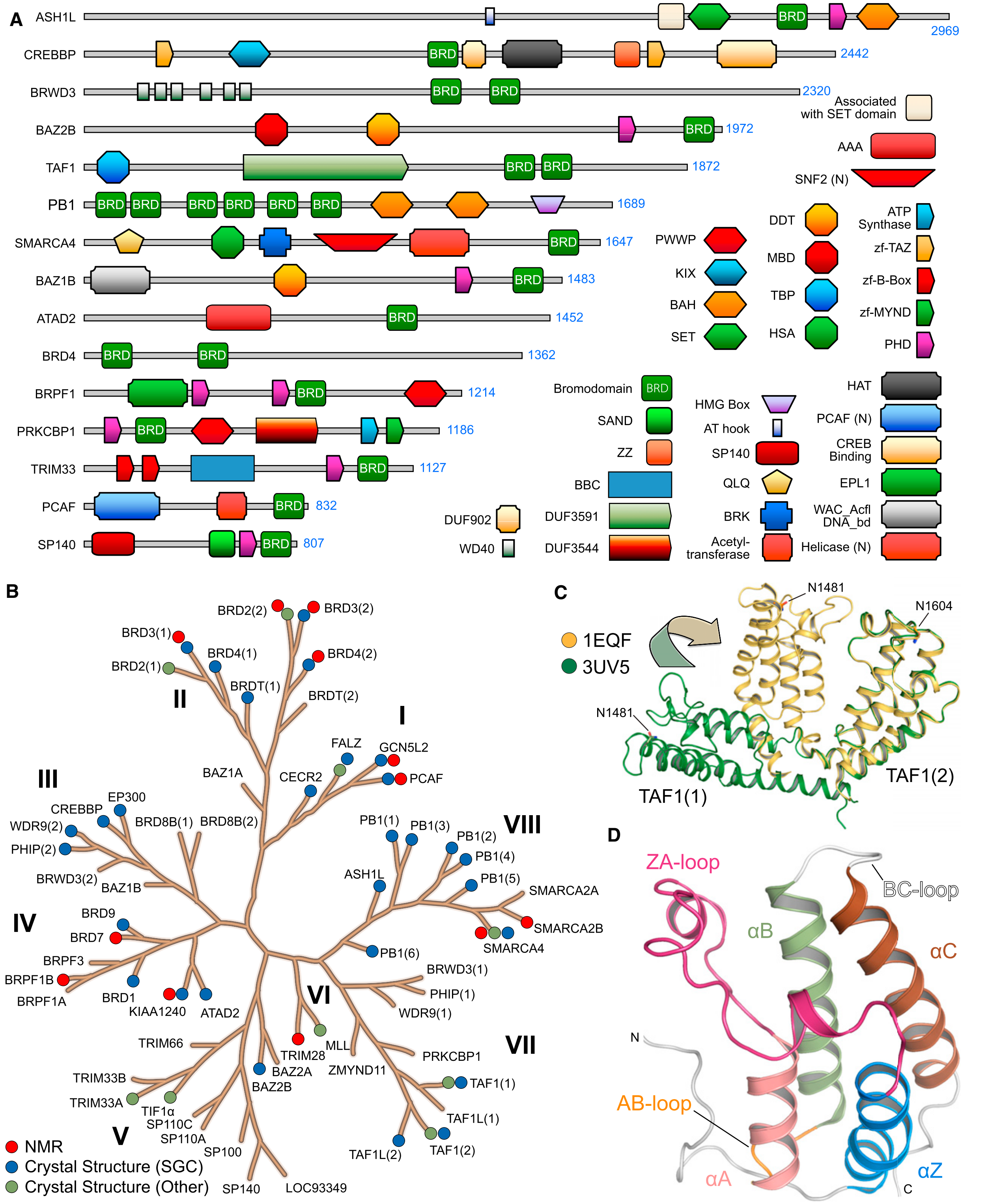

Figure 1. Domain Organization, Phylogenetic Tree, and Overall Fold of BRDs

(A) Domain organization of representative proteins that contain BRDs. The name and the length of the selected proteins are shown on the bar chart in the left panel. The positions of the different domains are highlighted as shown by the legend on the right.

(B) Phylogenetic tree of the human BRD family. The different families are named by Roman numbers (I–VIII). Structures determined in this study, by NMR, or by other groups are indicated by blue, red, and green dots, respectively.

Despite the low degree of overall sequence homology, all BRDs shared a conserved overall fold comprising four α helices (α_Z, α_A, α_B, α_C) linked by highly variable loop regions (ZA and BC loops) that form the docking site for interacting recognition motifs (Figures 1D and S2A). The C and N termini are highly diverse and may comprise additional helices that extend the canonical BRD fold (e.g., the sixth BRD of PB1 has an additional C-terminal helix) or largely extended kinked helices that are present as C- or N-terminal extensions (for instance in TAF1L or ATAD2). The four helices form a deep cavity that is extended by the two loop regions (ZA and BC loops), creating a largely hydrophobic K_{ac} binding pocket. The most notable structural difference within the BRD core fold is a hairpin insertion located between helix α_Z and the ZA loop that is present in all family VIII members. The proximity to the K_{ac} binding site suggests that this insert may play a role in recruitment of acetylated binding partners. Loop insertions are frequently found within the ZA loop, resulting in substantial differences in the rim region of the binding pocket. Hydrophobic residues in the ZA loop may contribute to protein instability and the low crystallization success rate observed in our work for BRDs of families VI and V. Indeed, in the recently published structure of the MLL tandem PHD-BRD module, a flexible insertion found in the MLL ZA loop was deleted in order to generate a more stable construct (Wang et al., 2010).

In stark contrast to the conserved fold of BRDs (Figure S3A), their surface properties are highly diverse. The electrostatic potential of the surface area around the K_{ac} binding site ranges from highly positively to strongly negatively charged, suggesting that BRDs recognize largely different sequences (Figure 2). Based on their surface properties, interactions with highly basic histones are not likely for BRDs with highly positive surfaces, as observed for instance for the third BRD of PB1.

Structural superimposition of 33 BRD crystal structures and 4 NMR models revealed conserved motifs throughout the folded protein domain. To refer to specific sites, we chose the first BRD of BRD4 as a reference sequence for numbering of residues (Figure S2A). The N-terminal helix α_Z is highly diverse, but it contains three conserved hydrophobic residues oriented toward the core of the helical bundle. This conserved motif follows the generic sequence $\phi_1x_1x_2(x_3)\phi_2x_3x_4x_5(x_6)\phi_3$, where ϕ_i are hydrophobic residues, and x_j represent any amino acid. The insertions at x_3 are present in the N-terminal domain of BET family members. Insertions x_6 are present in the C-terminal BRDs of TAF1 and TAF1L and possibly PRKCBP1 (Figures S2A and S2B). Helix α_Z is flanked by a diverse sequence region and a β hairpin insert present in all family VIII BRDs (Figures S1 and S3B). These diverse loop inserts are typically followed by a short helical segment in the ZA loop. The C terminus of the helical segment is stabilized by a highly conserved phenylalanine (F83 in BRD4(1)) that is deeply buried by hydrophobic residues present in helix α_C, bridging both sides of the helical bundle (Figure S2C). The ZA loop harbors also three conserved proline residues in addition to hydrophobic residues such as the conserved V87/Y97 pair that closely pack to hydrophobic residues present in α_C stabilizing the loop conformation. A conserved tyrosine (Y97) defines the N terminus of the ZA loop helix present in all BRDs except TRIM28 and the sixth BRD of PB1, which have unusually short ZA loops that have lost this structural element (Figures 3A and S2C).

Helix α_A is preceded by a Pϕ_1D motif (ϕ_1 is a hydrophobic residue). The conserved aspartate caps the helix α_A forming a hydrogen bond with a backbone amide. Also for this helix, the main sites of conservation are hydrophobic residues that contribute to the stability of the core of the structure (Figure 3B). The loop region AB contains a highly conserved tyrosine (Y119) that hydrogen bonds to a conserved aspartate (D128) located in helix α_B, presumably stabilizing the loop-helix fold. The long helix α_B shows a conserved pattern of the sequence ϕxxDϕxx$\phi\phi$xNϕxxY/F (Figure 3C). A conserved asparagine (N135) hydrogen bonds with the ZA loop backbone linking to this α_B loop region that is additionally stabilized by a small hydrophobic core formed around the conserved aromatic residue (Y139) preceding the K_{ac} docking residue (N140). An asparagine residue that anchors K_{ac} by formation of a critical hydrogen bond initiates the BC loop. Structural comparison suggested that this asparagine can be replaced by other hydrogen bond donors, such as threonine or tyrosine side chains. In MLL, however, an aspartate occupies this position suggesting that this domain either does not bind acetylated lysine residues or has a significantly different mechanism to recognize its target sequence. Similar to helix α_Z, the C-terminal helix α_C exhibits little sequence conservation apart from a number of hydrophobic core residues (Figure 3D). In summary, we have identified several highly conserved sequence motifs in BRDs that serve to stabilize the structural fold and conformation of loop regions flanking the K_{ac} binding pocket. An overview of the sequence conservation is shown in Figure S2D.

Interactions of BRDs with Histone Acetylation Sites

Histone tails are hot spots of PTMs that play key roles in regulation of transcription and all aspects of chromatin biology. However, to date, no systematic study has addressed binding specificity of reader domains. Here, we used SPOT peptide arrays that cover all possible K_{ac} sites of the human histones (H1.4, H2A, H2B, H3, and H4) in order to identify interaction sites for 33 representative BRDs. To distinguish between K_{ac}-dependent and independent binding, we also included all corresponding unmodified peptides. In general, affinities of K_{ac} for BRDs are low, suggesting that additional interaction domains may be required for higher affinity target-specific binding, in vivo. In some cases we observed K_{ac}-independent interaction of BRDs with nonacetylated control peptides. To date, it is not clear

(C) Domain flexibility as seen in the tandem BRD modules of TAF1 di-domain structure (orange PDB: 1EQF) and a new structure (green PDB: 3UV5), highlighting the ability of BRDs to adopt different relative orientations that may influence the recognition of their target sequences.
(D) Overall structure of the BRD4(1) BRD. N and C termini and secondary structure elements are labeled.
See also Figure S1.

Table 1. Human Bromodomain Family

Protein	Name	Alias	Protein Function	Subcellular Localization[a]	UniProt ID	Reference
ASH1L	ash1 (absent, small, or homeotic)-like	ASH1, KMT2H	Methyltransferase	N, C	Q9NR48	(Gregory et al., 2007)
ATAD2	Two AAA domain containing protein	ANCCA	Transcriptional regulator	N	Q6PL18	(Ciró et al., 2009)
BAZ1A	Bromodomain adjacent to zinc finger domain, 1A	ACF1, WALp1, WCRF180	Chromatin remodeling factor	N	Q9NRL2	(Racki et al., 2009)
BAZ1B	Bromodomain adjacent to zinc finger domain, 1B	WSTF, WBSCR9	Chromatin remodeling factor, transcriptional regulator	N	Q9UIG0	(Cavellán et al., 2006)
BAZ2A	Bromodomain adjacent to zinc finger domain, 2A	TIP5, WALp3	Transcriptional repressor	N, C	Q9UIF9	(Zhou et al., 2009)
BAZ2B	Bromodomain adjacent to zinc finger domain, 2B	WALp4	Unknown	N, C	Q9UIF8	(Jones et al., 2000)
BRD1	Bromodomain-containing protein 1	BRL, BRPF2	Transcriptional regulator, scaffold protein	N, C	O95696	(Ullah et al., 2008)
BRD2	Bromodomain-containing protein 2	FSH, RING3	Transcriptional regulator	N	P25440	(LeRoy et al., 2008)
BRD3	Bromodomain-containing protein 3	ORFX, RING3L	Transcriptional regulator	N	Q15059	(LeRoy et al., 2008)
BRD4	Bromodomain-containing protein 4	CAP, MCAP, HUNK1	Transcriptional regulator	N	O60885	(Yang et al., 2008)
BRD7	Bromodomain-containing protein 7	BP75, NAG4, CELTIX1	Transcriptional regulator	N	Q9NPI1	(Kaeser et al., 2008)
BRD8B	Bromodomain-containing protein 8 B	SMAP, SMAP2	Transcriptional regulator	N	Q9H0E9-2	(Cai et al., 2003)
BRD9	Bromodomain-containing protein 9		Unknown	N, C	Q9H8M2	NA
BRDT	Bromodomain-containing protein, testis specific	BRD6	Chromatin remodeling factor	N	Q58F21	(Morinière et al., 2009)
BRPF1	Bromodomain- and PHD finger-containing protein 1A	BR140, Peregrin	Transcriptional activator	N, C	P55201-1	(Laue et al., 2008)
BRPF3	Bromodomain- and PHD finger-containing protein, 3		Unknown	N	Q9ULD4	NA
BRWD3	Bromodomain-containing protein disrupted in leukemia	BRODL	JAK-STAT signaling	N, C	Q6RI45	(Müller et al., 2005)
CECR2	Cat eye syndrome chromosome region		Chromatin remodeling factor	N	Q9BXF3	(Fairbridge et al., 2010)
CREBBP	CREB-binding protein	CBP, KAT3A	Histone acetyl transferase	N	Q92793	(Kalkhoven, 2004)
EP300	E1A-binding protein p300	p300, KAT3B	Histone acetyl transferase	N	Q09472	(Kalkhoven, 2004)
FALZ	Fetal Alzheimer antigen	BPTF, FAC1	Transcription factor	N	Q12830	(Li et al., 2006)
GCN5L2	General control of amino acid synthesis 5-like 2	KAT2A, GCN5	Histone acetyl transferase	N	Q92830	(Yang et al., 1996)
KIAA1240	KIAA1240 protein	ATAD2B	Unknown	N	Q9ULI0	NA
LOC93349	SP140-like	SP140L	Unknown	U	Q13342	NA
MLL	Myeloid/lymphoid or mixed lineage leukemia (trithorax homolog, *Drosophila*)	HRX, TRX1, CXXC7, ALL-1	Histone methyl transferase	N	Q03164	(Dou et al., 2005)
PB1	Polybromo 1	PBRM1, BAF180	Chromatin remodeling factor	N, C	Q86U86	(Xue et al., 2000)

Table 1. *Continued*

Protein	Name	Alias	Protein Function	Subcellular Localization[a]	UniProt ID	Reference
PCAF	P300/CBP-associated factor	KAT2B	Histone acetyl transferase	N	Q92831	(Dhalluin et al., 1999)
PHIP	Pleckstrin homology domain-interacting protein	WDR11, ndrp	Insulin signaling	N	Q8WWQ0	(Podcheko et al., 2007)
PRKCBP1	Protein kinase C-binding protein 1	ZMYND8, RACK7	Transcriptional regulator	N	Q9ULU4	(Fossey et al., 2000)
SMARCA2	SWI/SNF-related matrix-associated actin-dependent regulator of chromatin a2	BRM, SNF2L2	Chromatin remodeling factor, Splicing regulator	N	P51531	(Harikrishnan et al., 2005)
SMARCA4	SWI/SNF-related matrix-associated actin-dependent regulator of chromatin a4	BRG1, SNF2L4, SNF2LB	Chromatin remodeling factor	N	P51532	(Rada-Iglesias et al., 2011)
SP100	Nuclear antigen Sp100		Transcriptional regulator	N, C	P23497	(Yordy et al., 2005)
SP110	Nuclear antigen Sp110 A, nuclear antigen Sp110 C	IPR1	Transcriptional regulator	N	Q9HB58	(Bloch et al., 2000)
SP140	SP140 nuclear body protein	LYSP100	Transcriptional regulator	N, C	Q13342	(Zong et al., 2000)
TAF1	TAF1 RNA polymerase II, TATA box-binding protein (TBP)-associated factor	TAFII250	Transcription initiation	N	P21675	(Wassarman and Sauer, 2001)
TAF1L	TAF1-like RNA polymerase II, TATA box-binding protein (TBP)-associated factor	TAF(II)210	Transcription initiation	N	Q8IZX4	(Wang and Page, 2002)
TIF1α	Transcriptional intermediary factor 1	TRIM24, PTC6, RNF82,	Transcriptional regulator	N, C	O15164	(Tsai et al., 2010)
TRIM28	Tripartite motif-containing 28	KAP1, RNF96, TIF1β	Transcriptional regulator	N	Q13263	(Rowe et al., 2010)
TRIM33	Tripartite motif-containing 33 A	PTC7, RFG7, TIF1γ	Control of transcription elongation	N	Q9UPN9	(Bai et al., 2010)
TRIM66	Tripartite motif-containing 66	TIF1δ	Transcriptional repressor	N	O15016	(Khetchoumian et al., 2004)
WDR9	WD repeat domain 9	BRWD1	Chromatin remodeling factor	N	Q9NSI6	(Huang et al., 2003)
ZMYND11	Zinc finger, MYND domain containing 11	BS69, BRAM1	Transcriptional repressor	N	Q15326	(Masselink and Bernards, 2000)

[a]Nuclear (N) or cytoplasmic (C).

whether BRDs participate in K_{ac}-independent protein interactions as it has been described for PHDs that recognize a broad variety of differently methylated, acetylated, and nonmodified peptides (Lan et al., 2007; Org et al., 2008; Tsai et al., 2010).

We identified 485 interactions of BRDs to histone peptides that depend on the presence of a single K_{ac} site (Figures 4 and S4; Table S6). The nuclear body protein SP140 as well as the related protein LOC93349 and PCAF showed nonspecific binding to most peptides. In contrast, the second, fourth, fifth, and sixth BRD of PB1, MLL, and TRIM28 interacted with only a few histone K_{ac} peptides. Also, a number of promiscuous sequences were identified, such as the H2AK36 and H2BK85-containing peptides that interacted with most BRDs. To validate the detected interactions and to obtain accurate binding constants in solution, we synthesized 53 singly acetylated peptides and determined binding constants by ITC (Table S7). We included also 14 peptides that did not bind to BRDs in the SPOT array. As expected these peptides did not show measurable interactions by ITC, suggesting that false negatives are not a major concern in the SPOT array study. Also in agreement with the array study, binding of 20 identified interacting peptides was confirmed by ITC experiments showing K_D values between 3 and ~300 μM. However, 16 peptides that were selected based on published recognition sites did not give rise to detectable interactions in the SPOT array and still exhibited K_D values between 10 and 730 μM by ITC. The detection limit of SPOT

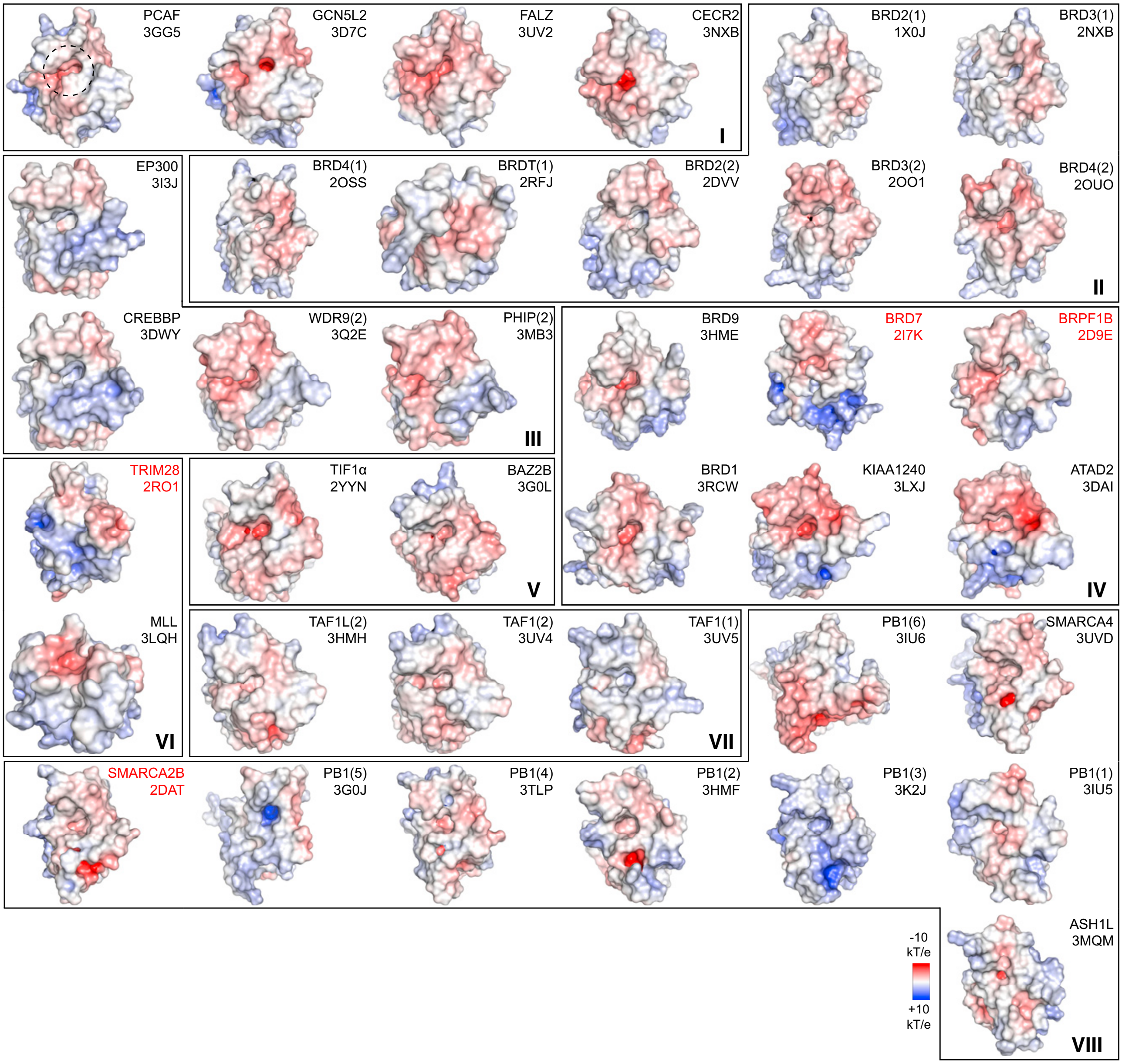

Figure 2. Electrostatic Surface Potential of Human BRDs

The domains are grouped into the eight BRD families (shown in roman numerals). Electrostatic surface potentials are shown between −10kT/e (red) and +10kT/e (blue). The BRD names and structures (PDB accession code in black for crystal structures and red for NMR models) are shown in the figure. All domains are shown in identical orientation with their acetyl-lysine binding site facing the reader and highlighted with a dashed circle on the top-left structure (PCAF). See also Figure S3.

arrays is about 500 μM, but the data suggest that SPOT arrays do not detect all possible interacting motifs. Steric constraints of the immobilized peptides and potentially the lack of sufficient N- and C-terminal flanking regions are the most likely reasons for the failure to detect BRD recognition motifs in SPOT arrays. In addition, 35 (12%) of acetyl-lysine containing peptides were not recognized by acetyl-lysine specific antibodies. However, most of these peptides contained proline residues in close proximity of the K_{ac} site, a likely reason for the failure of the antibody to recognize these sites. Other peptides showed crossreactivity with the His_6 antibody and have been removed from the analysis.

The false negative rate was particularly high for BET family members. Recently, it was demonstrated that murine BRDT preferentially recognized diacetylated motifs, whereas most monoacetylated peptides tested did not bind tightly to mBRDT BRDs (Morinière et al., 2009). This observation prompted us to design a systematic histone H3 array in which we explored combinations of acetylated and trimethylated (K_{me3}) lysines as

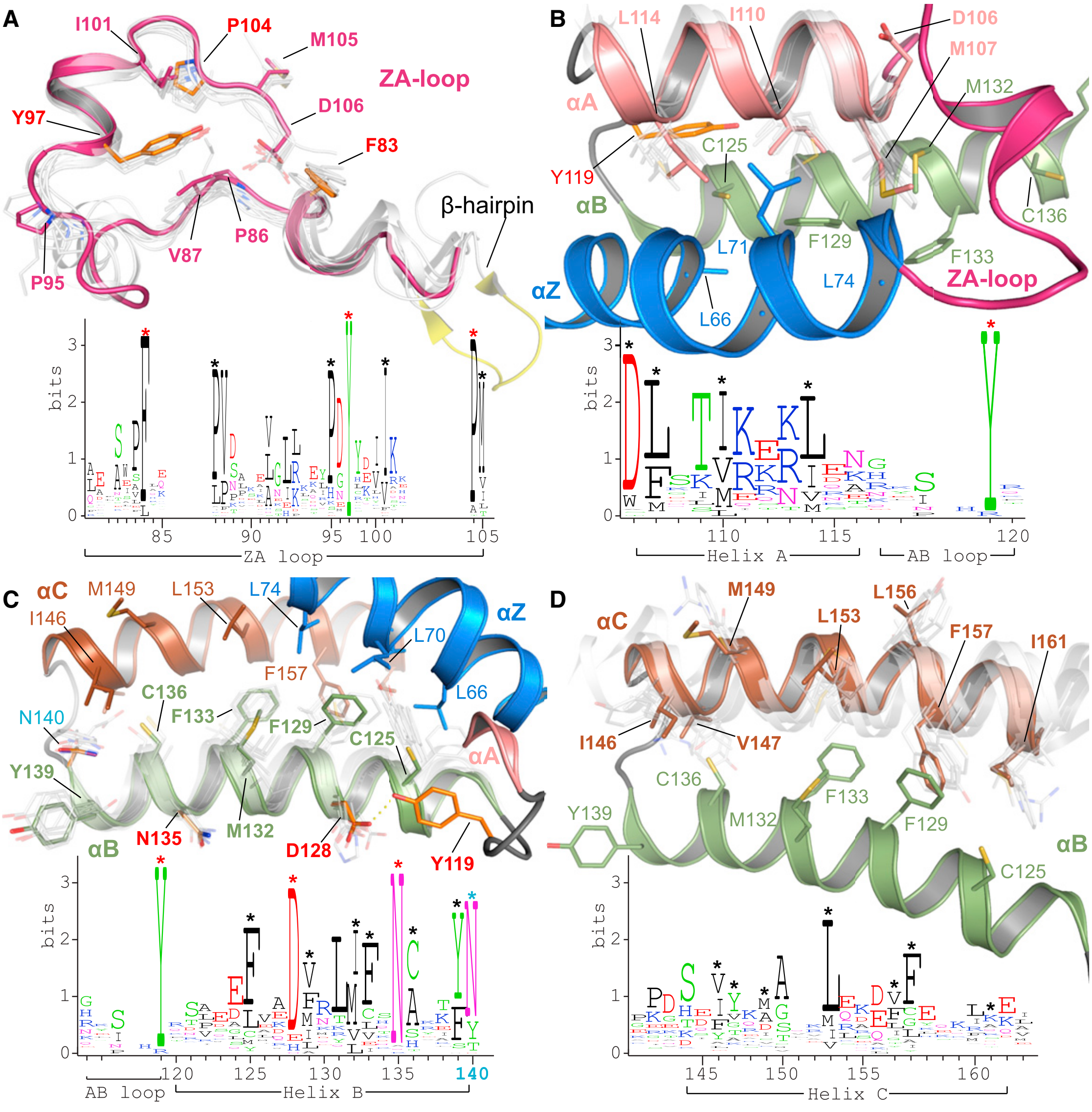

Figure 3. Conserved Residues and Sequence Logos

Conserved residues are shown as sequence logos (lower panel in each figure), and their location is shown in the ribbon diagram above. Secondary structure elements and residue labels are colored in all figures as follows: α_Z, blue; α_A, pink; α_B, green; α_C, brown; ZA loop, magenta. Motifs and location of residues are shown for (A) ZA loop, (B) helix α_A and AB loop, (C) AB loop and helix α_B, and (D) helix C. The conserved K_{ac} docking residue (N140) is shown in cyan. See also Figure S2.

well as phospho-serine/phospho-threonine (pS/pT) modifications around each acetylated lysine (Figures 5 and S5).

Interactions previously reported for singly acetylated lysine sites were largely confirmed. Interestingly, most of the 43 BRDs tested were highly sensitive to modifications flanking the K_{ac} mark. For instance, BRD4(2) did not interact with H3 peptides singly acetylated on K4. In contrast, this domain showed strong interaction with diacetylated H3 ($H3K4_{ac}K9_{ac}$) but not with the same peptide acetylated at K4 but trimethylated at K9 ($H3K4_{ac}K9_{me3}$). The strongest interaction was observed using diacetylated $H3K4_{ac}K9_{ac}$ in combination with phosphorylation at T3. Similarly, the BRD of FALZ showed no interaction with non- or singly acetylated K4 but interacted strongly with H3 $pT3K4_{ac}K9_{ac}$. Also, WDR9(2) and EP300 exclusively interacted with the triply modified H3 $pT3K4_{ac}K9_{ac}$ peptide. The WDR9(2) interaction with $H3K14_{ac}$ showed strong dependence on S10 and T11 phosphorylation as well as acetylation at K18. Indeed, ITC experiments showed that the binding affinity of many BRDs was significantly increased for multiply modified peptides (Tables S7 and S8). For example, the K_D of CREBBP decreased from 733 μM for $H3K14_{ac}$ to 131 μM for $H3pS10K14_{ac}K18_{ac}$ suggesting that many BRDs recognize a pattern of modifications rather than a single K_{ac} mark.

To obtain better insight into BRD recognition of multiply acetylated histone tails, we designed a systematic μ-SPOT

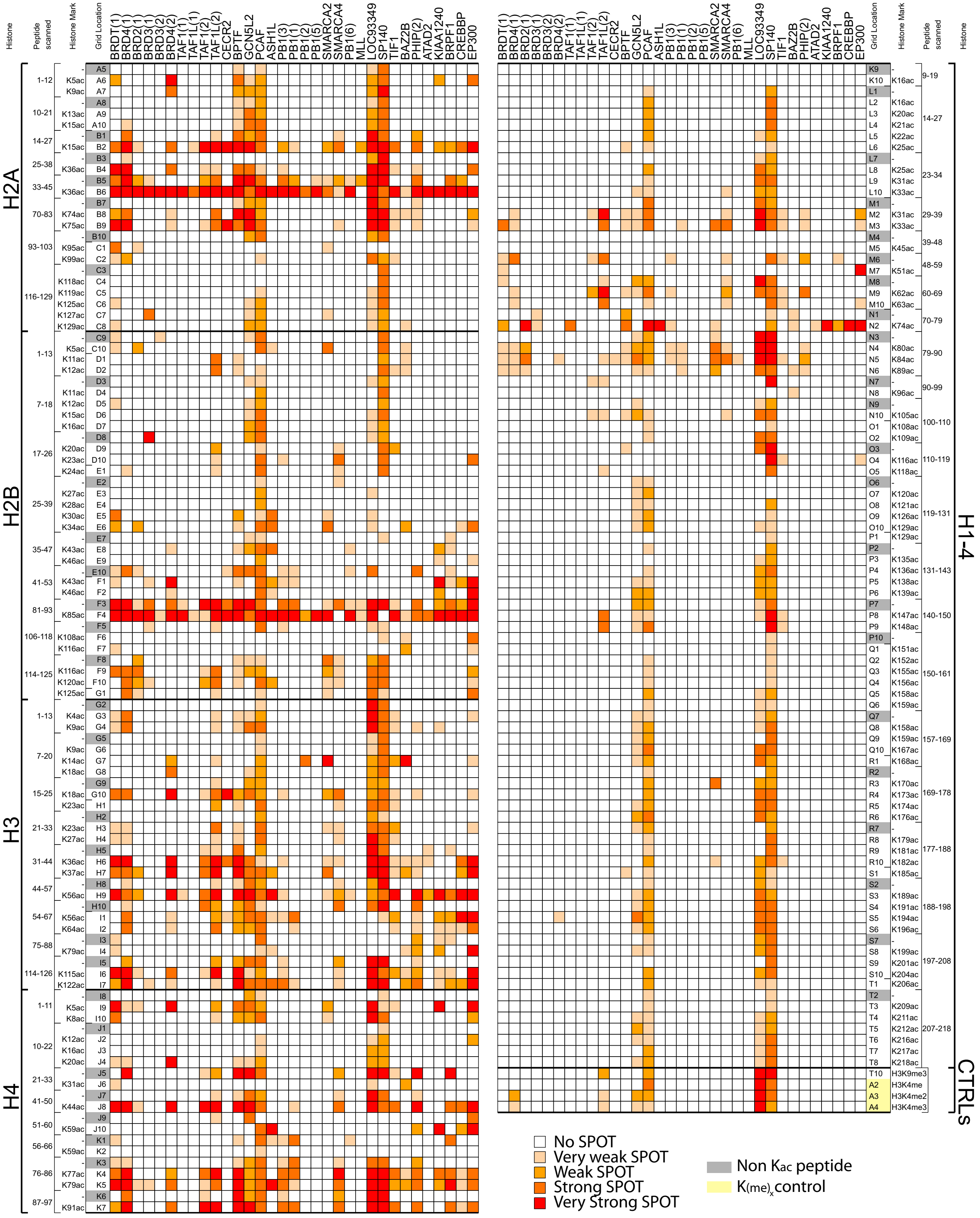

Histone
Peptide scanned
Histone Mark
Grid Location
BRDT(1)
BRD4(1)
BRD2(1)
BRD3(1)
BRD3(2)
BRD4(2)
TAF1(1)
TAF1L(1)
TAF1(2)
TAF1L(2)
CECR2
BPTF
GCN5L2
PCAF
ASH1L
PB1(3)
PB1(1)
PB1(2)
PB1(5)
SMARCA2
SMARCA4
PB1(6)
MLL
LOC93349
SP140
TIF1
BAZ2B
PHIP(2)
ATAD2
KIAA1240
BRPF1
CREBBP
EP300
H2A
H2B
H3
H4
H1-4
CTRLs
No SPOT
Very weak SPOT
Weak SPOT
Strong SPOT
Very Strong SPOT
Non Kac peptide
K(me)x control

array of peptide 11-mer that harbored multiple K_{ac} sites of the N-terminal tails of histones H3 and H4 (Figure 6A). Screening against BRDs of the BET family showed that BRD4(2) interacted with most combinations of two and three acetylated lysines, whereas BRD4(1) seemed to specifically recognize multiple marks found on the H4 tail. A tetra-acetylated H4 peptide that contained the acetylation sites K5, K8, K12, and K16 bound with single-digit micromolar K_D values to the first BRDs of BRD2 and BRD4, increasing affinity at least 20-fold when compared to single marks. The second BET BRDs bound to tetra-acetyl H4 peptides with about 10-fold weaker affinities, suggesting that the first BRD in BET proteins recognizes the H4 tail (Figure 6B). Recently, it was demonstrated that BRDT requires two K_{ac} residues for high-affinity binding (Morinière et al., 2009). Our peptide binding data suggest that the BET family and several other BRDs may also recognize multiply acetylated peptides. However, our binding data cannot discriminate between simultaneous recognition of two K_{ac} as opposed to increased avidity for a multiply modified peptide. In order to determine whether the diverse sequence and spacing of histone K_{ac} residues can be accommodated by a single BRD, we systematically determined cocrystal structures of BRD4(1) with the diacetylated peptides $H4K5_{ac}K8_{ac}$, $H4K12_{ac}K16_{ac}$, and $H4K16_{ac}K20_{ac}$. In all cases the two acetylated lysines bound simultaneously and with identical conformations to the BRD4(1) K_{ac} binding site (Figure 6C). The N-terminal K_{ac} always formed the anchoring hydrogen bond with the conserved asparagine (N140). In the N-terminal region of H4, flexible glycine residues allow variable peptide conformations with two ($H4_{1–11}K5_{ac}K8_{ac}$; Figure S6A) or three ($H4_{11–21}K12_{ac}K16_{ac}$; Figure S6B) linking residues, whereas the large side chains in $H4_{15–25}K16_{ac}K20_{ac}$ (Figure S6C) fit perfectly into surface grooves created by the ZA and BC loops. These structures explain the similar affinities observed for the various combinations of di- and triacetylated H4 peptides. They also suggest that the greater apparent affinity of BRD4(1) and BRD2(1) for tetra-acetylated H4 peptides is an avidity effect. However, not all diacetylated H4 sequences are compatible with this bidentate recognition process. The cocrystal structure of $H4_{7–17}K8_{ac}K12_{ac}$ with BRD4(1) revealed a canonical monoacetylated recognition mode (Figure S6D), suggesting that the $H4_{9–11}$ linker sequence is not suitable for a simultaneous recognition of the two K_{ac} by a single BRD. Consistent with this notion, ITC experiments revealed a binding stoichiometry (N) of 0.5, indicating binding of two BRDs to the $H4K8_{ac}K12_{ac}$ peptide, whereas only a single binding event with significantly increased affinity was observed for the $H4K5_{ac}$ $K8_{ac}$ peptide (Table S8). A representative set of ITC data is shown in Figure 6D.

We were interested in the sequence requirements of the diacetyl-lysine BET recognition and designed a systematic peptide array in which we modulated the spacer sequence and residue properties of residues located between the two K_{ac} binding sites (Figures 7A and S7). For the first BRDs of the BET family, a spacer of two glycine residues was optimal. However, BRD2(1) also tolerated longer linker sequences. For two-residue linkers, bulky amino acids in the first linker position were not tolerated, but changes of residue properties in the second linker position did not strongly influence binding. Intriguingly, the wild-type sequence "GG" of the H4 K5/K8 linker region seems highly optimized for interaction of the first BRD of BET family members. Binding of di-K_{ac} marks separated by three residue spacers as found in sequences linking the H4 K8/K16 and K16/K20 required a glycine or a hydrophobic residue in the first linker position for optimal binding to the first BET BRDs. Acidic residues in any linker position led to loss of interaction with H4 histone tail peptides. In contrast, the second BRDs of BET BRDs bound either weakly (BRD2), not at all (BRD3), or promiscuously (BRD4) to histone sequences and their variants present in this array. ITC data collected on the first BRD of BRD4 showed a 30-fold increase in affinity between the singly acetylated peptide $H4K5_{ac}$ to the most optimal wild-type peptide $H4K5_{ac}K8_{ac}$. In contrast, diacetylation had only a modest effect on binding affinities of the second BRD of BRD4. As reported for interactions with single acetylation sites in the case of the BRDs of BRD2 (Umehara et al., 2010), alanine mutants of the conserved asparagine (N140 and N443 in the first and second BRDs in BRD4) did also abolish binding of diacetylated peptides in both SPOT assays as well as in ITC (Figures 7B and 7C; Table S9).

In order to address the question of whether full-length BRD4 also interacts with the identified K_{ac} sites in the context of intact nucleosomes, we performed pull-down assays on nucleosomal preparations using Flag-tagged BRD4 and antibodies that specifically recognize K_{ac} sites. In agreement with our peptide array studies, we identified histone interaction of BRD4 with the H4 sites $K5_{ac}$, $K8_{ac}$, $K12_{ac}$, $K16_{ac}$, and H3 $K14_{ac}$ (Figure 7D). Unfortunately, no antibodies are currently available that specifically recognize diacetylated marks in histones H3 and H4. We therefore analyzed K_{ac}-enriched tryptic digests prepared from pull-downs of salt-extracted histone using C-terminally biotinylated BRD4(1) and BRD4(2) by mass spectroscopy (Figure 7E). We were able to detect numerous polyacetylated histone peptides associated with BRD4 BRDs; incubation with the active BRD4 inhibitor (+)-JQ1 (Filippakopoulos et al., 2010), but not its inactive stereoisomer (−)-JQ1, abrogated interaction with acetylated histones, indicating that the purifications were specific. Importantly, we observed that the first BRD, BRD4(1), interacted mostly with polyacetylated histone H4 peptides and that the majority of peptides identified contained at least two K_{ac} sites. These results are in good agreement with the strong increase in binding affinity and the preference for BRD4(1) for histone diacetyl marks observed in our in vitro binding studies.

Figure 4. Detected Interactions of BRDs with Histones in SPOT Arrays

A total of 33 BRDs were screened against an array of singly acetylated peptides that cover all possible acetylation sites in histones H1.4 (right panel), H2A, H2B, H3, and H4 (left panel). Non-K_{ac} specific interaction (corresponding nonacetylated) peptides are shaded in gray. Spots are shaded by different spot intensities as indicated in the figure. See also Figure S4.

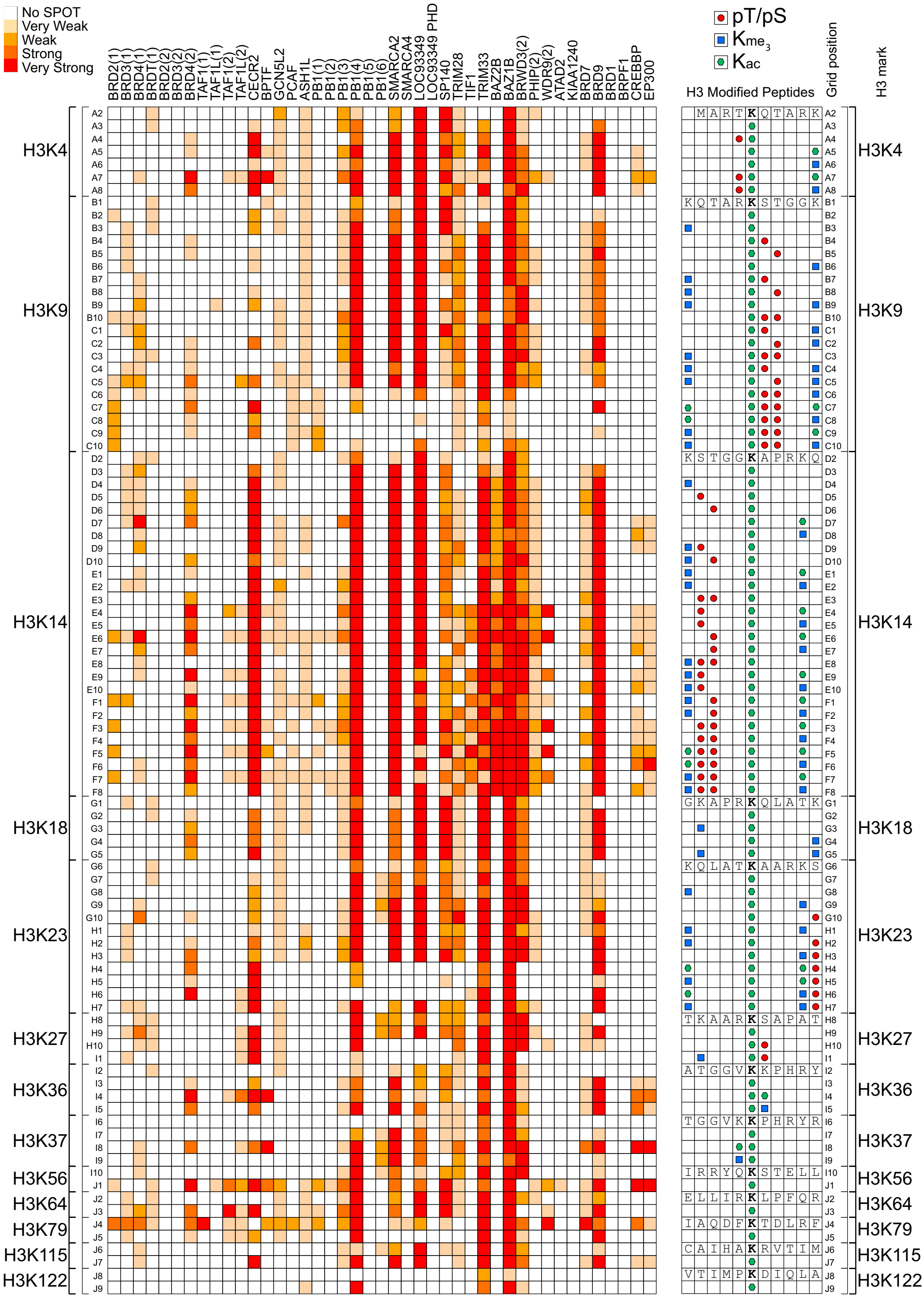

Figure 5. Influence of Neighboring PTMs on BRD Interaction with Histone H3

Shown are interactions detected in SPOT arrays shaded by spot intensity as indicated in the legend at the upper-left corner of the figure. The influence of lysine trimethylation (K_{me3}), acetylation, and phosphorylation (pT, pS) has been studied on binding to a central acetylated lysine epitope. The combination of the different modifications is indicated in the right panel. Nonmodified peptides have been included as controls to identify interactions independent of lysine acetylation. See also Figure S5.

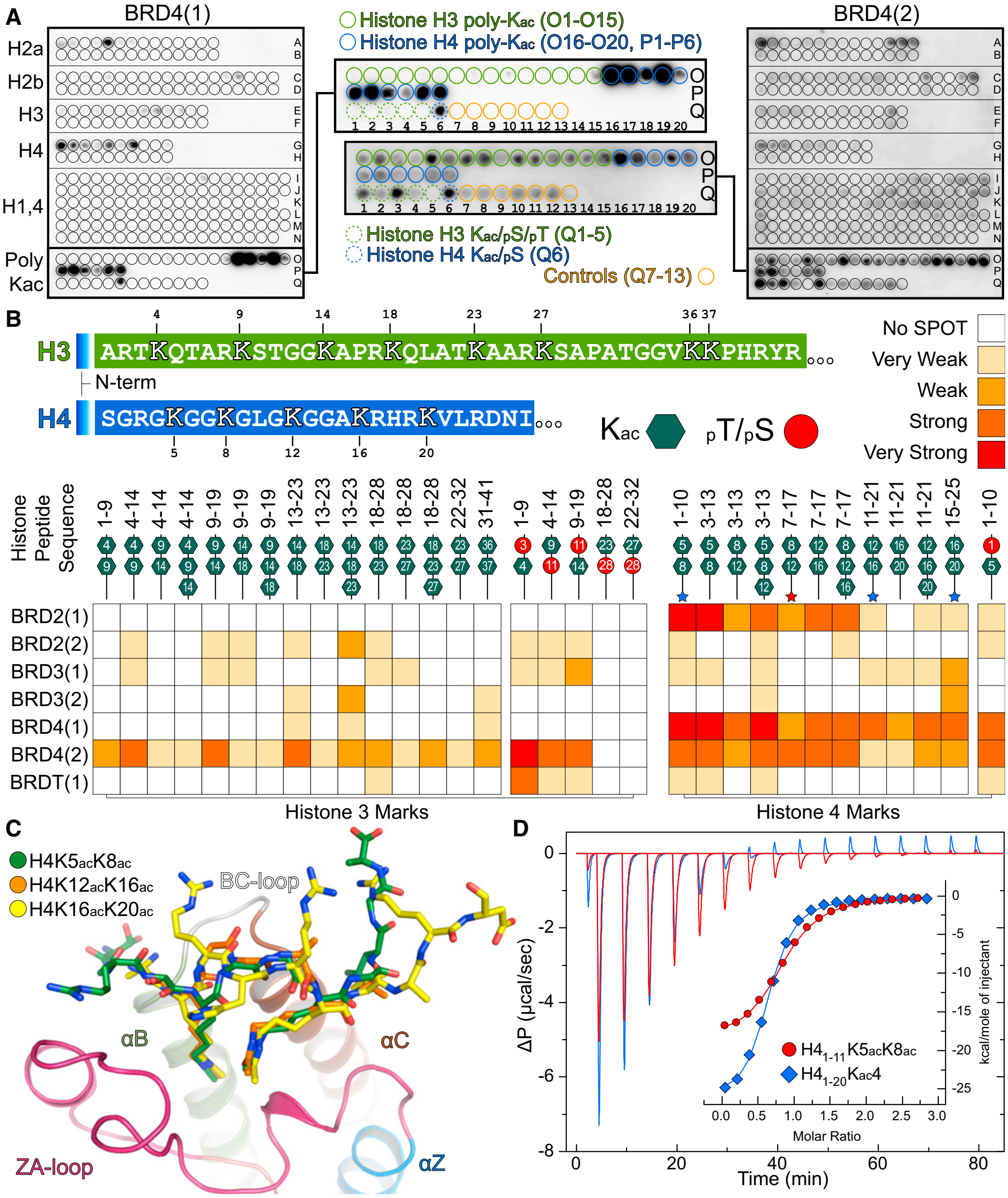

Figure 6. Binding of the N- and C-BRDs of BRD4 to Multiply Acetylated Histone H3 and H4

(A) Interactions detected in microSPOT arrays for BRD4(1) and BRD4(2) comprising multiply acetylated histone H3 (shown in green) or H4 (shown in blue) peptides.

(B) Peptide lengths are given together with the location of the K_{ac} marks (green hexagons). Binding of seven BRD members of the BET subfamily is summarized, highlighting the effect of multiple K_{ac} marks as well as neighboring Ser or Thr phosphorylation.

(C) Structural overlay of diacetylated peptides to the K_{ac} binding site of BRD4(1). The binding mode is retained, although the linker between marks and the flanking residues are not the same.

(D) Two representative ITC traces of polyacetylated histone H4 peptide binding to BRD4(1). Peptide sequences are shown in the inset.

See also Figure S6.

DISCUSSION

Recent developments in biotechnology and structural biology have facilitated rapid generation of structural data enabling determination of high-resolution structures of most members of certain protein families within a short time frame (Barr et al., 2009). The study presented here represents a comprehensive structural description of the entire human BRD family with at least one representative structural model for each branch in the BRD phylogenetic tree. Structural coverage of families I, II, and VIII is complete or nearly complete. These crystal structures enabled a detailed sequence comparison of this highly diverse

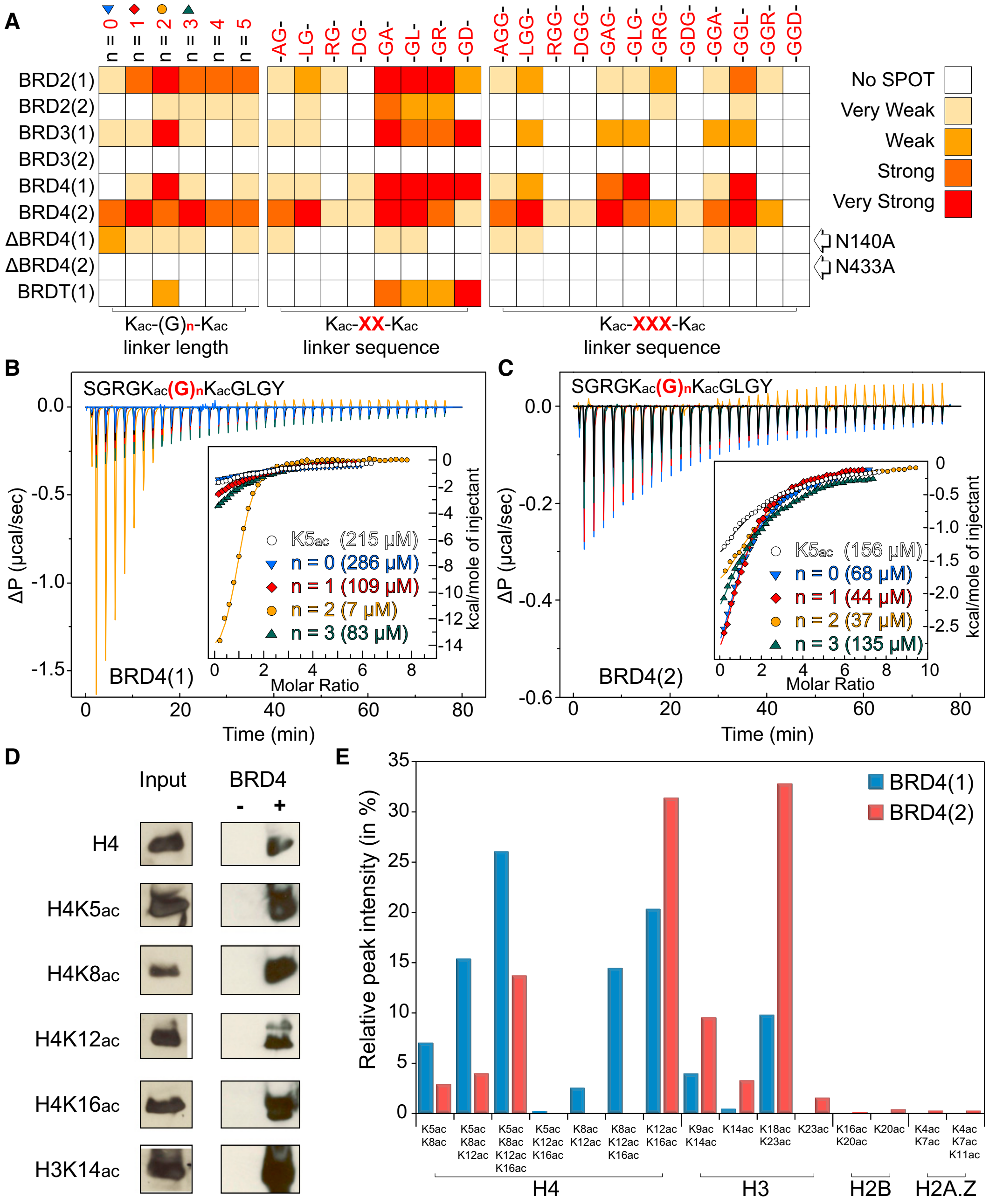

Figure 7. Effect of Distance between Acetylated Lysines and In Vivo Binding of BRD4

(A) Effect of poly-Gly and linker sequence on binding of doubly acetylated peptides to BET BRDs profiled by SPOT assays. Interactions are weakened or abolished when the docking asparagine is mutated to an Ala (N140A for the first and N433A for the second BRDs of BRD4).

(B) Effect of poly-Gly linker on the binding of H4K5$_{ac}$K8$_{ac}$ to BRD4(1) evaluated by ITC, demonstrating that the natural recognition sequence has the optimal sequence for binding. Peptide sequences are given in the inset.

(C) The second BRD of BRD4 is more promiscuous as demonstrated by ITC, exhibiting weaker binding for all tested peptides.

(D) Immunoprecipitation of Flag-tagged BRD4 from transfected cell nucleosome fraction and western blotting using anti-acetylated histone antibodies. Input represents 1% of total input. IgG was used for control immunoprecipitations.

(E) Individual BRD4 BRDs purify histones with distinct acetylation status from histone fractions. Acetylated histone peptides associated with biotinylated BRD4(1) or BRD4(2) were identified by LC-MS/MS, and the relative peak intensity of individual peptides was expressed as a ratio of peak area of the specific peptide to the sum of all peak areas for acetylated histone peptides in each sample.

See also Figure S7.

domain family. Importantly, although the protein family database Pfam (Finn et al., 2010) extended the BRD fold from the initially predicted central helices α_A and α_B (Haynes et al., 1992) to a 110 residue motif (Jeanmougin et al., 1997), sequence-based tools still fail to predict correct domain boundaries for BRDs that contain long ZA and BC loop insertions. The excellent structural coverage of the BRD family enabled the identification of BRD signature motifs and family-specific secondary structure elements, such as the ZA loop helix α_{AZ} and the subfamily VIII-specific β hairpin insert.

Probing BRD Histone Recognition by Peptide Arrays

Peptide arrays offer a rapid technology for screening protein-peptide interactions. The technology was developed more than a decade ago (Reineke et al., 2001) and has recently been applied to study epigenetic methyl-lysine reader domain interactions with histone tails (Nady et al., 2008, 2011). Recent progress in array technology allows peptide densities of up to 40,000 spots per square centimeter of solid support, enabling in principle genome-wide analysis of reader domains with peptidic recognition motifs (Beyer et al., 2009). To date, more than 100 histone PTMs that function as recruitment platforms for chromatin proteins have been described (Kouzarides, 2007). We chose, therefore, a systematic peptide array that covered all possible histone acetylation sites to characterize a representative set of BRD reader domains. However, many BRDs may interact with acetylation sites present in nonhistone proteins. In fact, we did not observe interaction with histone peptides for a number of BRDs.

Recognition sites for only a few BRDs have been previously characterized, and reported substrate affinities range from the low micromolar to the millimolar K_D range. Specific recognition sites in histones identified by our SPOT arrays that contained only a single K_{ac} site per peptide had binding affinities between 3 and 350 μM, which fall into the affinity range that has been reported for other BRD-K_{ac} interactions (Shen et al., 2007; Zeng et al., 2008). However, comparison of binding constants determined in solution by ITC with SPOT intensity did not always correlate, suggesting that peptides linked to cellulose supports used in this study did not allow quantification of binding affinities. However, a recent study found good correlation with SPOT intensities and substrate K_M values for deacetylases indicating improved correlation for proteins with enzymatic activity (Smith et al., 2011).

Many BRDs Recognize Patterns of PTMs

The weak contribution of the K_{ac} mark to the binding affinity of BRDs to their target sites makes BRD interactions particularly sensitive to changes in the environment of the K_{ac} site. The high density of PTMs in histones and other signaling molecules results in a large number of potential combinations of marks that regulate chromatin-templated recognition processes. In this study we selected a limited but systematic set of combinations that may be present in histone H3 and comprehensively profiled this array against the human BRD family. The observed strong influence of neighboring PTMs, such as phosphorylation, on recognition of their target sites by BRDs suggests tight coupling of phosphorylation signaling with epigenetic mechanisms of regulation. Many examples of this coupling have been reported for chromatin-modifying enzymes. For instance, H3S10 phosphorylation has been shown to be functionally linked to GCN5-mediated acetylation at H3K14 (Lo et al., 2000, 2001), and crosstalk of the three marks, H3K9$_{ac}$, H3pS10, and H4K16$_{ac}$, regulates transcriptional elongation of certain genes by providing a nucleosome platform that recruits BRD4/P-TEFb (Zippo et al., 2009). Also, H3pS10 is a prerequisite for H3K4 trimethylation (Li et al., 2011), which in turn has been shown to prevent phosphorylation at H3T3 by haspin (Eswaran et al., 2009). These data strongly suggest that combinatorial motifs rather than single PTMs determine the cellular outcome of processes regulated by epigenetic reader domains. This hypothesis would also explain the large amount of contradictory results in studies where single marks have been assigned specific function such as transcriptional activation or silencing. Thus, the reading process of the "histone code" is a sophisticated, nuanced chromatin language that recognizes combinations of marks rather than single PTMs (Berger, 2007).

Simultaneous Binding of Multiple Acetyl-Lysines to a Single BRD

Recent structural and biophysical studies demonstrated that murine BRDT requires at least two adjacent acetylation sites for tight interaction with the histone H4 tails (Morinière et al., 2009). Our SPOT array and ITC data showed that multiple K_{ac} sites are generally required for specific recognition of the histone H4 tail by all human BET family members.

We were interested if interactions of diacetyl-lysine also occur outside the BET BRD family. Using rigid docking of the H4K5$_{ac}$ K8$_{ac}$ peptide onto all available crystal structures revealed that a number of other BRDs would have an acetyl-lysine binding site architecture that would be compatible with the binding of this diacetylated peptide (data not shown).

The presence of multiple reader modules in chromatin modification complexes led to the proposal that distinct epigenetic signatures are interpreted by a multivalent reading process that engages diverse binding modules (Ruthenburg et al., 2007). For instance, recently, the dual-reader module PHD-BRD in BPTF has been shown to specifically recognize a combination of H4K16$_{ac}$ and H3K4$_{me3}$ at the mononucleosome level (Ruthenburg et al., 2011). Similarly, the tandem Tudor domain of UHRF1 recognizes H3 when K9 is trimethylated, and K4 is unmodified, a histone modification state associated with heterochromatin (Nady et al., 2011). Combinations of PHD and BRDs are particularly frequent and are a hallmark of BRD proteins in families V and VI, and the PHD-BRD structure showed that the two reader domains form a single, stable functional unit (Tsai et al., 2010). The work by Tsai et al. (2010) also suggests that the TRIM24 PHD-BRD di-domain binds two different histone tails in opposite orientations. Similarly, our array and ITC studies on BRD4 showed that the first BRD of this protein has high affinity for the histone H4 tail, whereas the second BRD most likely recognizes multiply acetylated marks in histone H3. This would be consistent with the notion that proteins that harbor multiple reader domains act as integration platforms for different chromatin proteins.

Epigenetic Reader Domains Are Promising Drug Targets

BRDs have recently emerged as promising targets for the development of protein interaction inhibitors (Chung et al., 2011; Filippakopoulos et al., 2010; Hewings et al., 2011; Nicodeme et al., 2010). The acetylation of lysine residues neutralizes the charge of the primary amine. As a consequence, BRD acetyl-lysine binding sites are deep and largely hydrophobic binding pockets that represent attractive targeting sites for the development of K_{ac} competitive inhibitors. Proteins containing epigenetic reader modules have been implicated in the development of many diseases (Baker et al., 2008; Muller et al., 2011; Reynoird et al., 2010).

The recent development of potent and highly specific K_{ac} competitive inhibitors for BET BRDs provides a compelling case for targeting these BRDs for the treatment of an extremely aggressive subtype of squamous cell carcinoma that is caused by chromosomal rearrangement of BRD3 or BRD4 with NUT (Filippakopoulos et al., 2010; French, 2010b). Recent data strongly suggested that targeting BET BRDs will be beneficial for many diverse cancer types due to downregulation of oncogenes such as c-Myc (Dawson et al., 2011; Delmore et al., 2011; Mertz et al., 2011). The structural data presented here provide the foundation for the rational design of selective BRD inhibitors that will be valuable tools for our understanding of the role of epigenetic reader modules in health and disease.

EXPERIMENTAL PROCEDURES

Protein Purification

BRD constructs were subcloned into pET28-derived expression vectors. All proteins were expressed as His_6-tagged fusions and were purified using Ni-chelating affinity chromatography. Analytical details for construct design, protein expression, and purification are given in the Extended Experimental Procedures and in Tables S1 and S2.

SPOT Assays

Peptides were synthesized on cellulose membranes using a MultiPep SPOT peptide arrayer (Intavis). His_6-tagged BRDs were added to a final concentration of 1 μM, and blots were developed using an ECL kit (Thermo Scientific) following the manufacturer's protocol.

ITC

Experiments were carried out on a VP-ITC or an ITC200 microcalorimeter (MicroCal, Northampton, MA, USA). In most cases a single binding site model was employed, supplied with the MicroCal Origin software package.

Crystallization and Structure Determination

Individual proteins were crystallized at either 4°C or 20°C, and X-ray diffraction data were collected on beamlines listed in Table S5. Structures were solved by molecular replacement and were refined as described in detail in the Extended Experimental Procedures. Crystallization conditions, data collection, refinement statistics, and PDB accession codes are listed in Tables S3, S4, and S5.

Histone Immunoprecipitation

HEK293 cells were grown in DMEM with 10% fetal bovine serum (FBS) and transfected with *BRD4*-Flag (UniProt: O60885, residues 1–1,362, cloned in pcDNA5) vector using GeneJuice (EMD) according to the manufacturer's instructions. Nucleosome isolation protocol was based on Ruthenburg et al. (2011) with modification as described in the Extended Experimental Procedures.

LC-MS/MS Analysis of Acetylated Histones and Their Quantitation

HeLa cells were grown in DMEM supplemented with 10% FBS and antibiotics (penicillin-streptomycin). Histones were extracted with high salt (Shechter et al., 2007) and incubated with recombinant biotinylated BRDs in the presence of (+)-JQ1 or (−)-JQ1 prior to purification on Strep-Tactin Sepharose beads. After elution of bound histones using trifluoroacetic acid (TFA) and sample lyophilization, trypsin digestion was performed, and trypsin was inhibited. Acetylated peptides were purified using anti-K_{ac} agarose beads (ImmuneChem Pharmaceuticals), and both the unbound fraction and the bound fraction (eluted in TFA) were prepared for mass spectrometry (MS). LC-MS/MS was performed using a NanoLC-Ultra 2D plus HPLC system (Eksigent) coupled to a LTQ-Orbitrap Velos (Thermo Electron) equipped with a nanoelectrospray ion source (Proxeon Biosystems). Spectra were assigned by Mascot (Matrix Science, v2.3) against the human RefSeq database (version 45). Relative quantitation of acetylated peptides was achieved with Proteome Discoverer 1.2 (Thermo Electron). The efficiency of purification with each BRD was monitored by analyzing the fraction unbound to anti-K_{ac}; specificity was ascertained by analyzing the samples incubated with the inhibitor (+)-JQ1.

ACCESSION NUMBERS

The models and structure factors of the reported bromodomain proteins have been deposited in the Protein Data Bank with the PDB accession codes 2NXB (BRD3(1)), 2OO1 (BRD3(2)), 2OSS (BRD4(1)), 2OUO (BRD4(2)), 2RFJ (BRDT(1)), 3HME (BRD9), 3D7C (GCN5L2), 3DAI (ATAD2), 3G0L (BAZ2B), 3GG3 (PCAF), 3DWY (CREBBP), 3UV2 (FALZ), 3IU5 (PB1(1)), 3HMF (PB1(2)), 3K2J (PB1(3)), 3TLP (PB1(4)), 3G0J (PB1(5)), 3IU6 (PB1(6)), 3I3J (EP300), 3HMH (TAF1L(2)), 3UV4 (TAF1(2)), 3LXJ (KIAA1240), 3MQM (ASH1L), 3MB3 (PHIP(2)), 3UVD (SMARCA4), 3NXB (CECR2), 3Q2E (WDR9(2)), 3UV5 (TAF1(1/2)), 3RCW (BRD1), 3UVW (BRD4(1)/H4K5$_{ac}$K8$_{ac}$), 3UVX (BRD4(1)/H4K12$_{ac}$K16$_{ac}$), 3UVY (BRD4(1)/H4K16$_{ac}$K20$_{ac}$), 3UW9 (BRD4(1)/H4K8$_{ac}$K12$_{ac}$), 3P1C (CREBBP/K_{ac}), 3P1D (CREBBP/N-Methyl-2-pyrrolidone), and 3MB4 (PB1(5)/N-Methyl-2-pyrrolidone).

SUPPLEMENTAL INFORMATION

Supplemental Information includes Extended Experimental Procedures, seven supplemental figures, and nine supplemental tables and can be found with this article online at doi:10.1016/j.cell.2012.02.013.

ACKNOWLEDGMENTS

The SGC is a registered charity (number 1097737) that receives funds from the Canadian Institutes for Health Research, the Canada Foundation for Innovation, Genome Canada, GlaxoSmithKline, Pfizer, Eli Lilly, the Novartis Research Foundation, the Ontario Ministry of Research and Innovation, and the Wellcome Trust. P.F. is supported by a Wellcome Trust Career Development Fellowship (095751/Z/11/Z). T.P. and A.-C.G. are supported by a Global Leadership Round in Genomics and Life Sciences from the Ontario Ministry of Health and Innovation. J.-P.L. is supported by a fellowship from the Natural Sciences and Engineering Research Council of Canada. A.-C.G. and C.H.A. hold Canada Research Chairs in Functional Proteomics and Structural Proteomics, respectively.

Received: July 5, 2011
Revised: October 16, 2011
Accepted: January 13, 2012
Published: March 29, 2012

REFERENCES

Bai, X., Kim, J., Yang, Z., Jurynec, M.J., Akie, T.E., Lee, J., LeBlanc, J., Sessa, A., Jiang, H., DiBiase, A., et al. (2010). TIF1gamma controls erythroid cell fate by regulating transcription elongation. Cell *142*, 133–143.

Baker, L.A., Allis, C.D., and Wang, G.G. (2008). PHD fingers in human diseases: disorders arising from misinterpreting epigenetic marks. Mutat. Res. *647*, 3–12.

Barr, A.J., Ugochukwu, E., Lee, W.H., King, O.N., Filippakopoulos, P., Alfano, I., Savitsky, P., Burgess-Brown, N.A., Müller, S., and Knapp, S. (2009). Large-scale structural analysis of the classical human protein tyrosine phosphatome. Cell *136*, 352–363.

Berger, S.L. (2007). The complex language of chromatin regulation during transcription. Nature *447*, 407–412.

Beyer, M., Block, I., König, K., Nesterov, A., Fernandez, S., Felgenhauer, T., Schirwitz, C., Leibe, K., Bischoff, R.F., Breitling, F., and Stadler, V. (2009). A novel combinatorial approach to high-density peptide arrays. Methods Mol. Biol. *570*, 309–316.

Bloch, D.B., Nakajima, A., Gulick, T., Chiche, J.D., Orth, D., de La Monte, S.M., and Bloch, K.D. (2000). Sp110 localizes to the PML-Sp100 nuclear body and may function as a nuclear hormone receptor transcriptional coactivator. Mol. Cell. Biol. *20*, 6138–6146.

Cai, Y., Jin, J., Tomomori-Sato, C., Sato, S., Sorokina, I., Parmely, T.J., Conaway, R.C., and Conaway, J.W. (2003). Identification of new subunits of the multiprotein mammalian TRRAP/TIP60-containing histone acetyltransferase complex. J. Biol. Chem. *278*, 42733–42736.

Cavellán, E., Asp, P., Percipalle, P., and Farrants, A.K. (2006). The WSTF-SNF2h chromatin remodeling complex interacts with several nuclear proteins in transcription. J. Biol. Chem. *281*, 16264–16271.

Choudhary, C., Kumar, C., Gnad, F., Nielsen, M.L., Rehman, M., Walther, T.C., Olsen, J.V., and Mann, M. (2009). Lysine acetylation targets protein complexes and co-regulates major cellular functions. Science *325*, 834–840.

Chung, C.W., Coste, H., White, J.H., Mirguet, O., Wilde, J., Gosmini, R.L., Delves, C., Magny, S.M., Woodward, R., Hughes, S.A., et al. (2011). Discovery and characterization of small molecule inhibitors of the BET family bromodomains. J. Med. Chem. *54*, 3827–3838.

Ciró, M., Prosperini, E., Quarto, M., Grazini, U., Walfridsson, J., McBlane, F., Nucifero, P., Pacchiana, G., Capra, M., Christensen, J., and Helin, K. (2009). ATAD2 is a novel cofactor for MYC, overexpressed and amplified in aggressive tumors. Cancer Res. *69*, 8491–8498.

Dawson, M.A., Prinjha, R.K., Dittmann, A., Giotopoulos, G., Bantscheff, M., Chan, W.I., Robson, S.C., Chung, C.W., Hopf, C., Savitski, M.M., et al. (2011). Inhibition of BET recruitment to chromatin as an effective treatment for MLL-fusion leukaemia. Nature *478*, 529–533.

Delmore, J.E., Issa, G.C., Lemieux, M.E., Rahl, P.B., Shi, J., Jacobs, H.M., Kastritis, E., Gilpatrick, T., Paranal, R.M., Qi, J., et al. (2011). BET bromodomain inhibition as a therapeutic strategy to target c-Myc. Cell *146*, 904–917.

Dhalluin, C., Carlson, J.E., Zeng, L., He, C., Aggarwal, A.K., and Zhou, M.M. (1999). Structure and ligand of a histone acetyltransferase bromodomain. Nature *399*, 491–496.

Dou, Y., Milne, T.A., Tackett, A.J., Smith, E.R., Fukuda, A., Wysocka, J., Allis, C.D., Chait, B.T., Hess, J.L., and Roeder, R.G. (2005). Physical association and coordinate function of the H3 K4 methyltransferase MLL1 and the H4 K16 acetyltransferase MOF. Cell *121*, 873–885.

Eswaran, J., Patnaik, D., Filippakopoulos, P., Wang, F., Stein, R.L., Murray, J.W., Higgins, J.M., and Knapp, S. (2009). Structure and functional characterization of the atypical human kinase haspin. Proc. Natl. Acad. Sci. USA *106*, 20198–20203.

Fairbridge, N.A., Dawe, C.E., Niri, F.H., Kooistra, M.K., King-Jones, K., and McDermid, H.E. (2010). Cecr2 mutations causing exencephaly trigger misregulation of mesenchymal/ectodermal transcription factors. Birth Defects Res. A Clin. Mol. Teratol. *88*, 619–625.

Filippakopoulos, P., Qi, J., Picaud, S., Shen, Y., Smith, W.B., Fedorov, O., Morse, E.M., Keates, T., Hickman, T.T., Felletar, I., et al. (2010). Selective inhibition of BET bromodomains. Nature *468*, 1067–1073.

Finn, R.D., Mistry, J., Tate, J., Coggill, P., Heger, A., Pollington, J.E., Gavin, O.L., Gunasekaran, P., Ceric, G., Forslund, K., et al. (2010). The Pfam protein families database. Nucleic Acids Res. *38* (Database issue), D211–D222.

Fossey, S.C., Kuroda, S., Price, J.A., Pendleton, J.K., Freedman, B.I., and Bowden, D.W. (2000). Identification and characterization of PRKCBP1, a candidate RACK-like protein. Mamm. Genome *11*, 919–925.

French, C.A. (2010a). Demystified molecular pathology of NUT midline carcinomas. J. Clin. Pathol. *63*, 492–496.

French, C.A. (2010b). NUT midline carcinoma. Cancer Genet. Cytogenet. *203*, 16–20.

French, C.A., Miyoshi, I., Aster, J.C., Kubonishi, I., Kroll, T.G., Dal Cin, P., Vargas, S.O., Perez-Atayde, A.R., and Fletcher, J.A. (2001). BRD4 bromodomain gene rearrangement in aggressive carcinoma with translocation t(15;19). Am. J. Pathol. *159*, 1987–1992.

Goodwin, G.H., and Nicolas, R.H. (2001). The BAH domain, polybromo and the RSC chromatin remodelling complex. Gene *268*, 1–7.

Gregory, G.D., Vakoc, C.R., Rozovskaia, T., Zheng, X., Patel, S., Nakamura, T., Canaani, E., and Blobel, G.A. (2007). Mammalian ASH1L is a histone methyltransferase that occupies the transcribed region of active genes. Mol. Cell. Biol. *27*, 8466–8479.

Harikrishnan, K.N., Chow, M.Z., Baker, E.K., Pal, S., Bassal, S., Brasacchio, D., Wang, L., Craig, J.M., Jones, P.L., Sif, S., and El-Osta, A. (2005). Brahma links the SWI/SNF chromatin-remodeling complex with MeCP2-dependent transcriptional silencing. Nat. Genet. *37*, 254–264.

Haynes, S.R., Dollard, C., Winston, F., Beck, S., Trowsdale, J., and Dawid, I.B. (1992). The bromodomain: a conserved sequence found in human, Drosophila and yeast proteins. Nucleic Acids Res. *20*, 2603.

Hewings, D.S., Wang, M., Philpott, M., Fedorov, O., Uttarkar, S., Filippakopoulos, P., Picaud, S., Vuppusetty, C., Marsden, B., Knapp, S., et al. (2011). 3,5-dimethylisoxazoles act as acetyl-lysine-mimetic bromodomain ligands. J. Med. Chem. *54*, 6761–6770.

Huang, H., Rambaldi, I., Daniels, E., and Featherstone, M. (2003). Expression of the Wdr9 gene and protein products during mouse development. Dev. Dyn. *227*, 608–614.

Huang, H., Zhang, J., Shen, W., Wang, X., Wu, J., Wu, J., and Shi, Y. (2007). Solution structure of the second bromodomain of Brd2 and its specific interaction with acetylated histone tails. BMC Struct. Biol. *7*, 57.

Jacobson, R.H., Ladurner, A.G., King, D.S., and Tjian, R. (2000). Structure and function of a human TAFII250 double bromodomain module. Science *288*, 1422–1425.

Jeanmougin, F., Wurtz, J.M., Le Douarin, B., Chambon, P., and Losson, R. (1997). The bromodomain revisited. Trends Biochem. Sci. *22*, 151–153.

Jones, D.T. (1999). Protein secondary structure prediction based on position-specific scoring matrices. J. Mol. Biol. *292*, 195–202.

Jones, M.H., Hamana, N., Nezu, J., and Shimane, M. (2000). A novel family of bromodomain genes. Genomics *63*, 40–45.

Kaeser, M.D., Aslanian, A., Dong, M.Q., Yates, J.R., III, and Emerson, B.M. (2008). BRD7, a novel PBAF-specific SWI/SNF subunit, is required for target gene activation and repression in embryonic stem cells. J. Biol. Chem. *283*, 32254–32263.

Kalkhoven, E. (2004). CBP and p300: HATs for different occasions. Biochem. Pharmacol. *68*, 1145–1155.

Kanno, T., Kanno, Y., Siegel, R.M., Jang, M.K., Lenardo, M.J., and Ozato, K. (2004). Selective recognition of acetylated histones by bromodomain proteins visualized in living cells. Mol. Cell *13*, 33–43.

Khetchoumian, K., Teletin, M., Mark, M., Lerouge, T., Cerviño, M., Oulad-Abdelghani, M., Chambon, P., and Losson, R. (2004). TIF1delta, a novel HP1-interacting member of the transcriptional intermediary factor 1 (TIF1) family expressed by elongating spermatids. J. Biol. Chem. *279*, 48329–48341.

Kouzarides, T. (2000). Acetylation: a regulatory modification to rival phosphorylation? EMBO J. *19*, 1176–1179.

Kouzarides, T. (2007). Chromatin modifications and their function. Cell *128*, 693–705.

Lan, F., Collins, R.E., De Cegli, R., Alpatov, R., Horton, J.R., Shi, X., Gozani, O., Cheng, X., and Shi, Y. (2007). Recognition of unmethylated histone H3 lysine 4 links BHC80 to LSD1-mediated gene repression. Nature *448*, 718–722.

Lane, A.A., and Chabner, B.A. (2009). Histone deacetylase inhibitors in cancer therapy. J. Clin. Oncol. *27*, 5459–5468.

Laue, K., Daujat, S., Crump, J.G., Plaster, N., Roehl, H.H., Kimmel, C.B., Schneider, R., Hammerschmidt, M., and Hammerschmidt, M.; Tübingen 2000 Screen Consortium. (2008). The multidomain protein Brpf1 binds histones and is required for Hox gene expression and segmental identity. Development *135*, 1935–1946.

LeRoy, G., Rickards, B., and Flint, S.J. (2008). The double bromodomain proteins Brd2 and Brd3 couple histone acetylation to transcription. Mol. Cell *30*, 51–60.

Li, H., Ilin, S., Wang, W., Duncan, E.M., Wysocka, J., Allis, C.D., and Patel, D.J. (2006). Molecular basis for site-specific read-out of histone H3K4me3 by the BPTF PHD finger of NURF. Nature *442*, 91–95.

Li, Y., Sun, L., Zhang, Y., Wang, D., Wang, F., Liang, J., Gui, B., and Shang, Y. (2011). The histone modifications governing TFF1 transcription mediated by estrogen receptor. J. Biol. Chem. *286*, 13925–13936.

Lo, W.S., Trievel, R.C., Rojas, J.R., Duggan, L., Hsu, J.Y., Allis, C.D., Marmorstein, R., and Berger, S.L. (2000). Phosphorylation of serine 10 in histone H3 is functionally linked in vitro and in vivo to Gcn5-mediated acetylation at lysine 14. Mol. Cell *5*, 917–926.

Lo, W.S., Duggan, L., Emre, N.C., Belotserkovskya, R., Lane, W.S., Shiekhattar, R., and Berger, S.L. (2001). Snf1—a histone kinase that works in concert with the histone acetyltransferase Gcn5 to regulate transcription. Science *293*, 1142–1146.

Masselink, H., and Bernards, R. (2000). The adenovirus E1A binding protein BS69 is a corepressor of transcription through recruitment of N-CoR. Oncogene *19*, 1538–1546.

Mertz, J.A., Conery, A.R., Bryant, B.M., Sandy, P., Balasubramanian, S., Mele, D.A., Bergeron, L., and Sims, R.J., III. (2011). Targeting MYC dependence in cancer by inhibiting BET bromodomains. Proc. Natl. Acad. Sci. USA *108*, 16669–16674.

Morinière, J., Rousseaux, S., Steuerwald, U., Soler-López, M., Curtet, S., Vitte, A.L., Govin, J., Gaucher, J., Sadoul, K., Hart, D.J., et al. (2009). Cooperative binding of two acetylation marks on a histone tail by a single bromodomain. Nature *461*, 664–668.

Müller, P., Kuttenkeuler, D., Gesellchen, V., Zeidler, M.P., and Boutros, M. (2005). Identification of JAK/STAT signalling components by genome-wide RNA interference. Nature *436*, 871–875.

Muller, S., Filippakopoulos, P., and Knapp, S. (2011). Bromodomains as therapeutic targets. Expert Rev. Mol. Med. *13*, e29.

Nady, N., Min, J., Kareta, M.S., Chédin, F., and Arrowsmith, C.H. (2008). A SPOT on the chromatin landscape? Histone peptide arrays as a tool for epigenetic research. Trends Biochem. Sci. *33*, 305–313.

Nady, N., Lemak, A., Walker, J.R., Avvakumov, G.V., Kareta, M.S., Achour, M., Xue, S., Duan, S., Allali-Hassani, A., Zuo, X., et al. (2011). Recognition of multivalent histone states associated with heterochromatin by UHRF1. J. Biol. Chem. *286*, 24300–24311.

Nicodeme, E., Jeffrey, K.L., Schaefer, U., Beinke, S., Dewell, S., Chung, C.W., Chandwani, R., Marazzi, I., Wilson, P., Coste, H., et al. (2010). Suppression of inflammation by a synthetic histone mimic. Nature *468*, 1119–1123.

Org, T., Chignola, F., Hetényi, C., Gaetani, M., Rebane, A., Liiv, I., Maran, U., Mollica, L., Bottomley, M.J., Musco, G., and Peterson, P. (2008). The autoimmune regulator PHD finger binds to non-methylated histone H3K4 to activate gene expression. EMBO Rep. *9*, 370–376.

Owen, D.J., Ornaghi, P., Yang, J.C., Lowe, N., Evans, P.R., Ballario, P., Neuhaus, D., Filetici, P., and Travers, A.A. (2000). The structural basis for the recognition of acetylated histone H4 by the bromodomain of histone acetyltransferase gcn5p. EMBO J. *19*, 6141–6149.

Podcheko, A., Northcott, P., Bikopoulos, G., Lee, A., Bommareddi, S.R., Kushner, J.A., Farhang-Fallah, J., and Rozakis-Adcock, M. (2007). Identification of a WD40 repeat-containing isoform of PHIP as a novel regulator of beta-cell growth and survival. Mol. Cell. Biol. *27*, 6484–6496.

Racki, L.R., Yang, J.G., Naber, N., Partensky, P.D., Acevedo, A., Purcell, T.J., Cooke, R., Cheng, Y., and Narlikar, G.J. (2009). The chromatin remodeller ACF acts as a dimeric motor to space nucleosomes. Nature *462*, 1016–1021.

Rada-Iglesias, A., Bajpai, R., Swigut, T., Brugmann, S.A., Flynn, R.A., and Wysocka, J. (2011). A unique chromatin signature uncovers early developmental enhancers in humans. Nature *470*, 279–283.

Reineke, U., Volkmer-Engert, R., and Schneider-Mergener, J. (2001). Applications of peptide arrays prepared by the SPOT-technology. Curr. Opin. Biotechnol. *12*, 59–64.

Reynoird, N., Schwartz, B.E., Delvecchio, M., Sadoul, K., Meyers, D., Mukherjee, C., Caron, C., Kimura, H., Rousseaux, S., Cole, P.A., et al. (2010). Oncogenesis by sequestration of CBP/p300 in transcriptionally inactive hyperacetylated chromatin domains. EMBO J. *29*, 2943–2952.

Rowe, H.M., Jakobsson, J., Mesnard, D., Rougemont, J., Reynard, S., Aktas, T., Maillard, P.V., Layard-Liesching, H., Verp, S., Marquis, J., et al. (2010). KAP1 controls endogenous retroviruses in embryonic stem cells. Nature *463*, 237–240.

Ruthenburg, A.J., Li, H., Patel, D.J., and Allis, C.D. (2007). Multivalent engagement of chromatin modifications by linked binding modules. Nat. Rev. Mol. Cell Biol. *8*, 983–994.

Ruthenburg, A.J., Li, H., Milne, T.A., Dewell, S., McGinty, R.K., Yuen, M., Ueberheide, B., Dou, Y., Muir, T.W., Patel, D.J., and Allis, C.D. (2011). Recognition of a mononucleosomal histone modification pattern by BPTF via multivalent interactions. Cell *145*, 692–706.

Shahbazian, M.D., and Grunstein, M. (2007). Functions of site-specific histone acetylation and deacetylation. Annu. Rev. Biochem. *76*, 75–100.

Shechter, D., Dormann, H.L., Allis, C.D., and Hake, S.B. (2007). Extraction, purification and analysis of histones. Nat. Protoc. *2*, 1445–1457.

Shen, W.Q., Xu, C., Huang, W., Zhang, J.H., Carlson, J.E., Tu, X.M., Wu, J.H., and Shi, Y.Y. (2007). Solution structure of human Brg1 bromodomain and its specific binding to acetylated histone tails. Biochemistry *46*, 2100–2110.

Smith, B.C., Settles, B., Hallows, W.C., Craven, M.W., and Denu, J.M. (2011). SIRT3 substrate specificity determined by peptide arrays and machine learning. ACS Chem. Biol. *6*, 146–157.

Tsai, W.W., Wang, Z., Yiu, T.T., Akdemir, K.C., Xia, W., Winter, S., Tsai, C.Y., Shi, X., Schwarzer, D., Plunkett, W., et al. (2010). TRIM24 links a non-canonical histone signature to breast cancer. Nature *468*, 927–932.

Ullah, M., Pelletier, N., Xiao, L., Zhao, S.P., Wang, K., Degerny, C., Tahmasebi, S., Cayrou, C., Doyon, Y., Goh, S.L., et al. (2008). Molecular architecture of quartet MOZ/MORF histone acetyltransferase complexes. Mol. Cell. Biol. *28*, 6828–6843.

Umehara, T., Nakamura, Y., Jang, M.K., Nakano, K., Tanaka, A., Ozato, K., Padmanabhan, B., and Yokoyama, S. (2010). Structural basis for acetylated histone H4 recognition by the human BRD2 bromodomain. J. Biol. Chem. *285*, 7610–7618.

Wang, P.J., and Page, D.C. (2002). Functional substitution for TAF(II)250 by a retroposed homolog that is expressed in human spermatogenesis. Hum. Mol. Genet. *11*, 2341–2346.

Wang, Z., Song, J., Milne, T.A., Wang, G.G., Li, H., Allis, C.D., and Patel, D.J. (2010). Pro isomerization in MLL1 PHD3-bromo cassette connects H3K4me readout to CyP33 and HDAC-mediated repression. Cell *141*, 1183–1194.

Wassarman, D.A., and Sauer, F. (2001). TAF(II)250: a transcription toolbox. J. Cell Sci. *114*, 2895–2902.

Xue, Y., Canman, J.C., Lee, C.S., Nie, Z., Yang, D., Moreno, G.T., Young, M.K., Salmon, E.D., and Wang, W. (2000). The human SWI/SNF-B chromatin-remodeling complex is related to yeast rsc and localizes at kinetochores of mitotic chromosomes. Proc. Natl. Acad. Sci. USA *97*, 13015–13020.

Yang, X.J., Ogryzko, V.V., Nishikawa, J., Howard, B.H., and Nakatani, Y. (1996). A p300/CBP-associated factor that competes with the adenoviral oncoprotein E1A. Nature *382*, 319–324.

Yang, Z., He, N., and Zhou, Q. (2008). Brd4 recruits P-TEFb to chromosomes at late mitosis to promote G1 gene expression and cell cycle progression. Mol. Cell. Biol. *28*, 967–976.

Yordy, J.S., Moussa, O., Pei, H., Chaussabel, D., Li, R., and Watson, D.K. (2005). SP100 inhibits ETS1 activity in primary endothelial cells. Oncogene *24*, 916–931.

Zeng, L., Zhang, Q., Gerona-Navarro, G., Moshkina, N., and Zhou, M.M. (2008). Structural basis of site-specific histone recognition by the bromodomains of human coactivators PCAF and CBP/p300. Structure *16*, 643–652.

Zhou, Y., Schmitz, K.M., Mayer, C., Yuan, X., Akhtar, A., and Grummt, I. (2009). Reversible acetylation of the chromatin remodelling complex NoRC is required for non-coding RNA-dependent silencing. Nat. Cell Biol. *11*, 1010–1016.

Zippo, A., Serafini, R., Rocchigiani, M., Pennacchini, S., Krepelova, A., and Oliviero, S. (2009). Histone crosstalk between H3S10ph and H4K16ac generates a histone code that mediates transcription elongation. Cell *138*, 1122–1136.

Zong, R.T., Das, C., and Tucker, P.W. (2000). Regulation of matrix attachment region-dependent, lymphocyte-restricted transcription through differential localization within promyelocytic leukemia nuclear bodies. EMBO J. *19*, 4123–4133.

EXTENDED EXPERIMENTAL PROCEDURES

Cloning

cDNA encoding human BRD containing proteins were obtained from different sources. Most of them were synthesized (Genscript) for codon optimization, some were provided by the MGC collection, the IMAGE collection or from commercial sources. The obtained cDNA sequences were used as templates to amplify BRD regions employing the Polymerase Chain Reaction (PCR) in the presence of Platinum Pfx DNA polymerase (Invitrogen, UK). All relevant details are listed in Table S1. PCR products were purified (QIAquick PCR Purification Kit, QIAGEN Ltd. UK) and further subcloned into pET28 derived expression vectors, pNIC28-Bsa4 (gi|124015065) or pNIC-CTHF (gi|124015079), using ligation independent cloning (Stols et al., 2002). Constructs were transformed into competent Mach1 cells (Invitrogen, UK) to yield the final plasmid DNA and were verified by sequencing.

Protein Expression and Purification for Biophysical Characterization

Constructs were transformed into competent BL21 (DE3) cells (Invitrogen) or into BL21 (DE3)-R3-pRARE2 cells (phage-resistant derivative with a pRARE plasmid encoding rare codon tRNAs). Cells were grown at 37°C either in Luria-Bertani medium (LB-broth, Merck) or in Terrific Broth (Merck) from overnight cultures. Protein expression was induced overnight with 0.1 mM isopropyl-β-D-thiogalactopyranoside (IPTG) at 18°C at an OD_{600} nm of 0.9 or 3.0 respectively. Cultures were harvested by centrifugation (8,700 x *g* for 15 min at 4°C) on a Beckman Coulter Avanti J-20 XP centrifuge, and then re-suspended in lysis buffer (50 mM HEPES, pH 7.5 at 20°C, 500 mM NaCl, 5 mM Imidazole, 5% glycerol and 0.5 mM tris(2-carboxyethyl)phosphine (TCEP) in the presence of 1:200 (v/v) Protease Inhibitor Cocktail III (Calbiochem). Cells were lysed at 4°C using an EmulsiFlex-C5 high pressure homogenizer (Avestin - Mannheim, Germany) and the DNA was removed by precipitation on ice for 30 min with 0.15% (v/v) of PEI (Polyethyleneimine). Lysates were cleared by centrifugation (16,000 x *g* for 1h at 4°C, JA 25.50 rotor, on a Beckman Coulter Avanti J-20 XP centrifuge) and were applied to a Nickel affinity column (nickel nitrilotriacetic acid (Ni-NTA) resin, QIAGEN Ltd., 5 ml, equilibrated with 20 ml lysis buffer). Columns were washed once with 30 ml of lysis buffer then twice with 10 ml of lysis buffer containing 30 mM Imidazole. Proteins were eluted using a step elution of imidazole in lysis buffer (50, 100, 150, 2 × 250 mM Imidazole). All fractions were collected and monitored by SDS-polyacrylamide gel electrophoresis (Bio-Rad Criterion Precast Gels, 4%–12% Bis-Tris, 1.0 mm, from Bio-Rad, CA. Gel run conditions: 180 V, 400 mA, 55 min in XT MES buffer). The eluted proteins were treated overnight at 4°C with TEV (Tobacco Etch Virus) protease to remove the hexa-histidine expression tag and were further purified by size exclusion chromatography on Superdex 75 16/60 HiLoad gel filtration columns (GE/Amersham Biosciences) on ÄktaPrime plus systems (GE/Amersham Biosciences). Proteins in 10 mM HEPES, 500 mM NaCl and 5% glycerol were concentrated to 10 mg/ml with a Amicon® Ultra (MILLIPORE) concentrators employing 10 kDa cut-offs (10 MWCO), flash frozen in liquid nitrogen and stored at −80°C. Protein concentration was estimated using a NanoDrop ND-1000 spectrophotometer (also see Table S2).

Protein Expression and Purification for Spot Assays

Soluble BRD constructs were expressed in LB media (5 × 50 ml) in the presence of 50 μg/ml kanamycin. Cell growth was allowed at 37°C to an optical density of about 0.5 (OD_{600nm}). Protein expression was induced by 1 mM IPTG, overnight, at 18°C. Cells were harvested by centrifugation at 4,000 × *g* for 15 min at 4°C and re-suspended in 4 ml of binding buffer (50 mM HEPES pH7.5 at 20°C; 500 mM NaCl; 5% glycerol, 5 mM Imidazole) complemented with 0.5 mM TCEP and 1:200 (v/v) Protease Inhibitor Cocktail III (Calbiochem). Cells were disrupted with an ultrasonic processor (SONICS Vibra-Cell, amplitude 60, 10 s ON, 10 s OFF, for 2 min). Lysates were cleared by centrifugation and were applied to Ni-NTA columns (QIAGEN Ltd., 2 ml, equilibrated with 20 ml lysis buffer). Columns were washed once with 30 ml of lysis buffer then twice with 10 ml of the same buffer containing 30 mM Imidazole. Proteins were eluted using a step elution of imidazole in lysis buffer (50, 100, 150, 2 × 250 mM Imidazole). All fractions were collected and monitored by SDS-polyacrylamide gel electrophoresis. Fractions containing the recombinant proteins were pooled and concentrated using Amicon® Ultra (MILLIPORE) concentrators (10 MWCO) to a final volume of 1 ml before being loaded on NAP-10 column (GE-Healthcare) in order to exchange the buffer to 25 mM HEPES (pH 7.5 at 20°C), 150 mM NaCl and 5% glycerol. Samples were flash frozen in liquid nitrogen and stored at −80°C until used (Table S2).

SPOT Assays

Three different types of SPOT membrane were utilized in order to probe preferences of BRD recognition sites: the first array contained peptides from all four core histones (H2A, H2B, H3 and H4) covering all possible lysine acetylation sites, with peptide sizes ranging from 10 to 14 amino acids. The second array contained short peptides (11 residues) from human histone 3 exploiting possible posttranslational modifications around each acetyl-lysine epitope, including phosphorylation (on serine and threonine residues), acetylation (on lysine residues) and trimethylation (on lysine residues). The third array covered all possible lysine acetylation sites found on the four core human histones, as well as peptides containing multiple adjacent acetyl-lysine epitopes, employing a micro-SPOT (μSPOT) technology using a smaller foot-print.

Membranes Synthesis

Peptides for the first two arrays were synthesized directly on cellulose membranes (Intavis) using a MultiPep SPOT peptide arrayer (Intavis) and commercially available standard (Intavis) or modified (Bachem) L-amino acid precursors as previously described (Nady

et al., 2008). The quality of the synthesized array was evaluated as follows: i) immune reactive peptides were identified by incubation of the membrane with an anti-His antibody in the absence of any His_6-tagged BRD. 8 peptides were recognized by this antibody and were excluded from the analysis.

In all cases a general monoclonal primary antibody against acetylated lysine (#9681, Cell Signaling Technology) was used to probe the proper incorporation of acetylated lysine on each membrane, using the protocol provided by the manufacturer for Western blotting. Phosphorylation was probed using a primary antibody against phosphorylated Ser10 (ab47297, Abcam) and Thr11 (ab5168, Abcam), employing an anti-rabbit HRP fragment secondary antibody (Amersham Biosciences).

Protein-Peptide Interaction Assay

Membranes were washed 3 × 5 min with PBST (3.2 mM Na_2HPO_4, 0.5 mM KH_2PO_4, 1.3 mM KCl, 135 mM NaCl and 0.1% Tween 20, pH 7.4) and were subsequently blocked with 5% milk in PBST overnight at 4°C in order to minimize nonspecific binding of the proteins to the membranes. After 2 washes with PBST (5 min each) followed by a single wash with PBS (3.2 mM Na_2HPO_4, 0.5 mM KH_2PO_4, 1.3 mM KCl, 135 mM NaCl pH 7.4) for 5 min, his_6 tagged BRD proteins were added to a final concentration of 1 μM and the membranes were incubated over night at 4°C in PBS. Each membrane was washed 3 times in PBST, blocked for 1 hr with 10% milk in PBST, and washed again 3 × 5 min with PBST. HPR-conjugated anti-His-tag antibodies (Novagen #71841) were added in 5% milk/PBST solution at a dilution of 1:2000. After 1 hr incubation, membranes were washed 3 × 20 min in PBST. The assay was developed with an ECL kit (Pierce ECL Western Blotting substrate, Thermo Scientific) following the manufacturer's protocol. Chem-illuminescence was detected with an Image reader (Fujifilm LAS-4000 ver.2.0) with an incremental exposure time of 5 min for a total of 80 min. Intensities of the resulting spots were quantified with the Kodak 1D ver.3.6.2 Scientific Imaging System. All experiments were performed at room temperature.

Membrane Stripping

Membranes were washed 3 × 10 min in water, 2 × 30 min in stripping buffer A (6 M Guanidinium HCl, 1% Triton X-100) followed by an overnight incubation with Stripping Buffer A and Talon Beads at room temperature. The next day each membrane was washed twice with Stripping Buffer B (500 mM Imidazole, 500 mM NaCl, 20mM TRIS-HCl, pH 7.5) for 30 min each, followed by a series of washes with deionized and distilled water (ddH_2O) at room temperature and at 60°C. Finally, a series of washes was performed, 10 min each with ddH_2O, 10% TFA (trifluoroacetic acid), ddH_2O, 20% EtOH, 50% EtOH and 95% EtOH. Membrane were dried overnight and stored for extended periods of time at −20°C until they were re-used.

Crystallization and Structure Determination

Individual proteins were crystallized in sitting drops at either 4°C or 20°C. Crystals were cryo-protected, flash frozen and X-ray diffraction data were collected at 100 K on beam lines X10SA at the Swiss Light Source (SLS), at Diamond (beam lines I02, I03, I04, I04.1), or at a Rigaku FRE Superbright home source. Diffraction images were indexed, and integrated using MOSFLM (Leslie and Powell, 2007), HKL2000 or XDS (Kabsch, 2010b) and data were scaled using SCALA (Evans, 2007), SCALEPACK (Otwinowski and Minor, 1997), or XSCALE (Kabsch, 2010a), respectively. Structures were solved by molecular replacement using PHASER (McCoy et al., 2005) and were refined against maximum likelihood targets using REFMAC (Murshudov et al., 1997). Iterative rounds of refinement were interspersed with manual rebuilding in COOT (Emsley and Cowtan, 2004). Thermal motions were analyzed using TLSMD (Painter and Merritt, 2006) and hydrogen atoms were included in late refinement cycles. Crystallization conditions, data collection and refinement statistics, PDB accession codes are compiled in Table S5.

Structure-Based Alignment and Sequence Analysis

Multiple sequence/structural alignments were carried out using STRAP (Gille and Frömmel, 2001) and ICM Pro (MolSoft LLC version 3.7-2c) (Abagyan et al., 1994) and were further manually edited (Figure S1). In the absence of an X-Ray or NMR structure model the PFAM boundaries for each BRD were extended using PSIPRED (version 2) (Jones, 1999) for secondary structure prediction and the secondary structure elements were further used to guide the STRAP/ICM alignment. A phylogenetic tree of the resulting structure based alignment was generated using the ClustalW2 program (Larkin et al., 2007) and is given in Figure 1B. Sequence conservations were visualized using the WebLogo (Crooks et al., 2004) online web server.

Isothermal Titration Calorimetry

Experiments were carried out on a VP-ITC microcalorimeter or an ITC200 (MicroCal, LLC Northampton, MA). All experiments were performed at 10 or 15°C in 50 mM HEPES pH 7.5, 150 mM NaCl. All titrations were conducted using an initial injection of 2 μl followed by 29 identical injections of 8 μl (VP-ITC) or 0.3 μl followed by identical injections of 1 μl (ITC200). The dilution heats were measured on separate experiments and were subtracted from the titration data. Thermodynamic parameters were calculated using $\Delta G = \Delta H - T\Delta S = -RT\ln K_B$, where ΔG, ΔH and ΔS are the changes in free energy, enthalpy and entropy of binding respectively. In most cases a single binding site model was employed, supplied with the MicroCal Origin software package. Multiple binding events were also confirmed with the software package SEDPHAT (Houtman et al., 2007). Binding constants and thermodynamic parameters are given in Tables S7 (Single K_{ac} marks), S8 (multiple marks) and S9 (linker sequences).

Histone Immunoprecipitation

HEK293 cells were grown in DMEM with 10% FBS and transfected with Brd4-Flag (UniProt: O60885, residues 1-1362, cloned in pcDNA5) vector using GeneJuice (EMD) according to the manufacturer's instructions. The transfected cells were treated with 500 nM (+)-JQ1 or (-)-JQ1 (Filippakopoulos et al., 2010) for 16 hr and cells collected by scraping. Nucleosome isolation protocol was based on Ruthenburg and co-workers (Ruthenburg et al., 2011) with modification at the end of the nuclease treatment. Briefly the cells were washed in buffer A (10 mM HEPES pH 7.9, 10 mM KCl, 1.5 mM $MgCl_2$, 340 mM sucrose, 10% (v/v) glycerol, protease inhibitors (Roche) TSA 1 μg/ml, beta-mercaptoethanol 5 mM. The cell pellet was resuspended in buffer A with 0.1% Triton x-100 and incubated on ice for 10 min. The nuclei were pelleted, washed twice with buffer A and resuspended in buffer A to nucleic acid concentration of 1.2 μg/μl measured as previously described (Brand et al., 2008). $CaCl_2$ was added to 2 mM, followed by the micrococcal nuclease (Worthington) (1 U/50 μg DNA) and the reaction incubated at 37°C for 10 min. The reaction stopped adding 4 mM EGTA on ice. To facilitate the release of digested nucleosomes, NaCl was added to final concentrations of 200 mM and reactions spun down at 13,000 rpm for 5 min. The soluble nucleosomes in the supernatant were collected and diluted (1:5) in the IP buffer (100 mM KCl 5% glycerol, 10 mM TRIS-HCl pH 8.0, 10 mg/ml BSA, protease inhibitors, 1 μg/ml TSA, beta-mercaptoethanol 5 mM). Anti-Flag M2 antibody (Sigma) or normal mouse IgG (Abcam) and protein G Dynabeads (Invitrogen) were added and incubated at 4°C overnight. The reactions were washed 5 times in the IP buffer and eluted with 100 mM TRIS-HCl pH 8.0, 150 mM NaCl, 2 mM EDTA, 5 mM DTT, 1% Triton, 3% SDS 15 mM beta-mercaptoethanol. The immunoprecipitated proteins were run on Tris-Bis PAGE (Invitrogen) and transferred to nitrocellulose membranes (Pall) that were probed with the following antibodies: $H4K5_{ac}$ (Abcam 61236), $H4K12_{ac}$ (Upstate 07-595), H4 (Abcam 7311), $H3K9_{ac}$ (Abcam 12179), $H4K16_{ac}$ (Active Motif 39167), $H4K18_{ac}$ (Cell Signaling Technology 2594) and secondary HRP conjugated antibodies (Cell Signaling Technology).

Preparation of Asynchronous and M Phase HeLa Cells

HeLa cells were grown in DMEM supplemented with 10% fetal bovine serum and antibiotics (Penicillin-Streptomycin cocktail). In order to generate cells arrested in M phase, cells at ~50% confluency were first treated with 2 mM thymidine for 24 h; the media was then removed, the cells washed with 1X PBS, and fresh media added for three hours to allow release from the thymidine block. Nocodazole (100 ng/ml) was then added for 16 hr to arrest cells in M phase. The cells were then harvested by "mitotic shake-off" to harvest rounded mitotic cells which were then washed with cold PBS and frozen as a dry pellet at −80°C.

High-Salt Extraction of Histones

Histones were extracted from asynchronous or M phase cells using high-salt as previously described (Shechter et al., 2007) with minor modifications. Briefly, frozen cell pellets from 20 × 15 cm plates of HeLa cells (corresponding to approximately 4×10^8 cells) were resuspended in 10 ml of cold extraction buffer (10 mM HEPES-NaOH pH 8.0, 10 mM KCl, 1.5 mM $MgCl_2$, 0.34 M sucrose, 10% glycerol, 0.1 mM PMSF, 10 mM sodium butyrate) with 0.2% NP-40 and incubated on ice for 10 min with occasional mixing. Samples were then centrifuged (6,500 x *g*, 5 min at 4°C) and the supernatant discarded. An additional 10 ml of cold extraction buffer without NP-40 was used to resuspend the pellet, and the mixture was centrifuged (6,500 x *g*, 5 min at 4°C). The supernatant was completely removed and the pellet resuspended in 10 ml of no-salt buffer (3 mM EDTA, 0.2 mM EGTA), vortexed intermittently for 2 min and further incubated at 4°C on a nutator for 30 min. The samples were then centrifuged (6,500 x *g*, 5 min at 4°C) and the supernatant discarded. Histones were extracted by vortexing the pellet in 10 ml of high-salt solubilization buffer (50 mM TRIS-HCl pH 8.0, 2.5 M NaCl, 0.5% NP-40) for 2 min, followed by incubation at 4°C on a nutator for 30 min. DNA was pelleted by centrifugation (16,000 x *g*, 10 min at 4°C) and the supernatant containing histones transferred to a fresh tube. Three buffer exchanges were performed with a spin filter device of 5,000 Da molecular weight cut-off to reduce salt concentration before storing at −80°C until further use.

Biotin-Tagged Bromodomain Affinity Purification

500 μg of salt-extracted histones from asynchronous or M phase HeLa cells were mixed with 50 μg of recombinant biotinylated BRD4(1) or BRD4(2) and incubated at 4°C on a nutator for 60 min in the presence of 1 μM (+)-JQ1 or 1 μM (-)-JQ1. Samples were transferred to a fresh tube containing 30 μl of streptactin sepharose beads (IBA BioTAGnology) and incubated for 60 min at 4°C on a nutator. The beads were then washed five times with 1 ml of wash buffer (50 mM HEPES-NaOH pH 8.0, 500 mM KCl, 2 mM EDTA, 0.1% NP-40, 10% glycerol) and two times with 1 ml of no-salt wash buffer (20 mM TRIS-HCl pH 8.0, 2 mM $CaCl_2$). Bound histones were eluted by incubating the beads in 1 ml of 0.5% Trifluoroacetic acid (TFA) at 4°C on a nutator. The supernatants were transferred to fresh tubes and then evaporated to dryness and stored at −80°C.

Protein Digestion and Acetylated Peptide Purification

Dried sample were resuspended in 100 μl of 20 mM TRIS-HCl pH 8.0, and 1 μg of trypsin (Sigma-Aldrich; Singles) was added to each sample. Samples were incubated overnight at 37°C with agitation, and supplemented with an extra 0.5 μg of trypsin before another incubation of 4 hr. The samples were then boiled for 10 min to inhibit trypsin activity and subsequently let to cool down to room temperature for approximately 15 min. Samples were then diluted to 400 μl with peptide wash buffer solution (50 mM MOPS pH 7.2, 10 mM $NaPO_4$, 50 mM NaCl) and incubated with 30 μl of anti-acetyl-lysine agarose beads (ImmuneChem Pharmaceuticals Inc.) overnight at 4°C. The next morning, the beads were collected by gentle centrifugation and the supernatant (unbound fraction)

transferred to a fresh tube for subsequent analysis by mass spectrometry. The beads were washed once with 1 ml of peptide wash buffer solution and once with 1 ml of no-salt wash buffer (20 mM TRIS-HCl pH 8.0, 2 mM $CaCl_2$). Peptides were eluted by incubating the beads with 1 ml of 0.5% TFA for 30 min at 4°C and then transferred to a fresh tube before being evaporated to dryness.

LC-MS/MS Analysis of Acetylated Histones and Their Quantitation

Dried peptides were dissolved in 5% formic acid and analyzed by LC-MS/MS using a NanoLC-Ultra 2D plus HPLC system (Eksigent, Dublin, USA) coupled to a LTQ-Orbitrap Velos (Thermo Electron, Bremen, Germany) equipped with a nanoelectrospray ion source (Proxeon Biosystems, Odense, Denmark). The LTQ-Orbitrap Velos instrument under Xcalibur 2.0 was operated in the data dependent mode to automatically switch between MS and up to 10 subsequent MS/MS acquisition. Raw MS and MS/MS spectra were processed using Prohits (Liu et al., 2010). Peptides and proteins were identified using the Mascot software (Matrix Science, London, UK) and the human RefSeq database (version 45, released on February 2nd 2010, containing 34,604 sequences). Mass tolerance of 7 ppm in MS mode and 0.6 Da in MS/MS mode with trypsin specificity were used, and 4 missed cleavage sites were allowed. No fixed modification was selected, but N-acetyl protein, N-pyroglutamine, oxidized methionine, acetylation of lysine and phosphorylation of serine, threonine and tyrosine were searched as variable modifications. Relative quantitation of acetylated peptides using MS spectra was achieved with Proteome Discoverer 1.2 (Thermo Electron, Bremen, Germany). The peak area for acetylated peptides co-purifying with BRD4(1) or BRD4(2) were calculated first by summing the areas under the curve for a given acetylated peptide in the asynchronous or nocodazole-arrested cells (for a given bromodomain), then by computing the area of all acetylated peptides associated with either bromodomain. The relative peak intensity of each acetylated peptide was then determined by expressing (in percent) the ratio of the area under the peak of a given peptide peak over the total area under the peak of all peptides. The efficiency of purification with each bromodomain was monitored by analyzing the fraction unbound to the anti-acetyl lysine beads; specificity in the interaction was ascertained by analyzing by quantitative MS each of the samples incubated with the inhibitors (-)-JQ1 and (+)-JQ1.

SUPPLEMENTAL REFERENCES

Abagyan, R., Totrov, M., and Kuznetsov, D. (1994). ICM—a new method for protein modeling and design: applications to docking and structure prediction from the distorted native conformation. J. Comput. Chem. *15*, 488–506.

Akai, Y., Adachi, N., Hayashi, Y., Eitoku, M., Sano, N., Natsume, R., Kudo, N., Tanokura, M., Senda, T., and Horikoshi, M. (2010). Structure of the histone chaperone CIA/ASF1-double bromodomain complex linking histone modifications and site-specific histone eviction. Proc. Natl. Acad. Sci. USA *107*, 8153–8158.

Brand, M., Rampalli, S., Chaturvedi, C.P., and Dilworth, F.J. (2008). Analysis of epigenetic modifications of chromatin at specific gene loci by native chromatin immunoprecipitation of nucleosomes isolated using hydroxyapatite chromatography. Nat. Protoc. *3*, 398–409.

Charlop-Powers, Z., Zeng, L., Zhang, Q., and Zhou, M.M. (2010). Structural insights into selective histone H3 recognition by the human Polybromo bromodomain 2. Cell Res. *20*, 529–538.

Crooks, G.E., Hon, G., Chandonia, J.M., and Brenner, S.E. (2004). WebLogo: a sequence logo generator. Genome Res. *14*, 1188–1190.

Emsley, P., and Cowtan, K. (2004). Coot: model-building tools for molecular graphics. Acta Crystallogr. D Biol. Crystallogr. *60*, 2126–2132.

Evans, P. (2007). SCALA - scale together multiple observations of reflections (Cambridge: MRC Laboratory of Molecular Biology).

Filippakopoulos, P., Qi, J., Picaud, S., Shen, Y., Smith, W.B., Fedorov, O., Morse, E.M., Keates, T., Hickman, T.T., Felletar, I., et al. (2010). Selective inhibition of BET bromodomains. Nature *468*, 1067–1073.

Gille, C., and Frömmel, C. (2001). STRAP: editor for STRuctural Alignments of Proteins. Bioinformatics *17*, 377–378.

Houtman, J.C., Brown, P.H., Bowden, B., Yamaguchi, H., Appella, E., Samelson, L.E., and Schuck, P. (2007). Studying multisite binary and ternary protein interactions by global analysis of isothermal titration calorimetry data in SEDPHAT: application to adaptor protein complexes in cell signaling. Protein Sci. *16*, 30–42.

Jacobson, R.H., Ladurner, A.G., King, D.S., and Tjian, R. (2000). Structure and function of a human TAFII250 double bromodomain module. Science *288*, 1422–1425.

Jones, D.T. (1999). Protein secondary structure prediction based on position-specific scoring matrices. J. Mol. Biol. *292*, 195–202.

Kabsch, W. (2010a). Integration, scaling, space-group assignment and post-refinement. Acta Crystallogr. D Biol. Crystallogr. *66*, 133–144.

Kabsch, W. (2010b). Xds. Acta Crystallogr. D Biol. Crystallogr. *66*, 125–132.

Klosi, E., Saro, D., and Spaller, M.R. (2007). Bivalent peptides as PDZ domain ligands. Bioorg. Med. Chem. Lett. *17*, 6147–6150.

Larkin, M.A., Blackshields, G., Brown, N.P., Chenna, R., McGettigan, P.A., McWilliam, H., Valentin, F., Wallace, I.M., Wilm, A., Lopez, R., et al. (2007). Clustal W and Clustal X version 2.0. Bioinformatics *23*, 2947–2948.

Leslie, A.G.W., and Powell, H. (2007). MOSFLM (Cambridge: MRC Laboratory of Molecular Biology).

Li, H., Ilin, S., Wang, W., Duncan, E.M., Wysocka, J., Allis, C.D., and Patel, D.J. (2006). Molecular basis for site-specific read-out of histone H3K4me3 by the BPTF PHD finger of NURF. Nature *442*, 91–95.

Liu, G., Zhang, J., Larsen, B., Stark, C., Breitkreutz, A., Lin, Z.Y., Breitkreutz, B.J., Ding, Y., Colwill, K., Pasculescu, A., et al. (2010). ProHits: integrated software for mass spectrometry-based interaction proteomics. Nat. Biotechnol. *28*, 1015–1017.

McCoy, A.J., Grosse-Kunstleve, R.W., Storoni, L.C., and Read, R.J. (2005). Likelihood-enhanced fast translation functions. Acta Crystallogr. D Biol. Crystallogr. *61*, 458–464.

Mujtaba, S., He, Y., Zeng, L., Farooq, A., Carlson, J.E., Ott, M., Verdin, E., and Zhou, M.M. (2002). Structural basis of lysine-acetylated HIV-1 Tat recognition by PCAF bromodomain. Mol. Cell *9*, 575–586.

Mujtaba, S., He, Y., Zeng, L., Yan, S., Plotnikova, O., Sachchidanand, Sanchez, R., Zeleznik-Le, N.J., Ronai, Z., and Zhou, M.M. (2004). Structural mechanism of the bromodomain of the coactivator CBP in p53 transcriptional activation. Mol. Cell *13*, 251–263.

Murshudov, G.N., Vagin, A.A., and Dodson, E.J. (1997). Refinement of macromolecular structures by the maximum-likelihood method. Acta Crystallogr. D Biol. Crystallogr. *53*, 240–255.

Nady, N., Min, J., Kareta, M.S., Chédin, F., and Arrowsmith, C.H. (2008). A SPOT on the chromatin landscape? Histone peptide arrays as a tool for epigenetic research. Trends Biochem. Sci. *33*, 305–313.

Otwinowski, Z., and Minor, W. (1997). Processing of X-ray diffraction data collected in oscillation mode. In Macromolecular Crystallography, Part A, C.W. Carter, Jr., and R. Sweet, eds. (San Diego: Academic Press Inc), pp. 307–326.

Painter, J., and Merritt, E.A. (2006). Optimal description of a protein structure in terms of multiple groups undergoing TLS motion. Acta Crystallogr. D Biol. Crystallogr. *62*, 439–450.

Ruthenburg, A.J., Li, H., Milne, T.A., Dewell, S., McGinty, R.K., Yuen, M., Ueberheide, B., Dou, Y., Muir, T.W., Patel, D.J., and Allis, C.D. (2011). Recognition of a mononucleosomal histone modification pattern by BPTF via multivalent interactions. Cell *145*, 692–706.

Shechter, D., Dormann, H.L., Allis, C.D., and Hake, S.B. (2007). Extraction, purification and analysis of histones. Nat. Protoc. *2*, 1445–1457.

Singh, M., Popowicz, G.M., Krajewski, M., and Holak, T.A. (2007). Structural ramification for acetyl-lysine recognition by the bromodomain of human BRG1 protein, a central ATPase of the SWI/SNF remodeling complex. ChemBioChem *8*, 1308–1316.

Stols, L., Gu, M.Y., Dieckman, L., Raffen, R., Collart, F.R., and Donnelly, M.I. (2002). A new vector for high-throughput, ligation-independent cloning encoding a tobacco etch virus protease cleavage site. Protein Expr. Purif. *25*, 8–15.

Sun, H., Liu, J., Zhang, J., Shen, W., Huang, H., Xu, C., Dai, H., Wu, J., and Shi, Y. (2007). Solution structure of BRD7 bromodomain and its interaction with acetylated peptides from histone H3 and H4. Biochem. Biophys. Res. Commun. *358*, 435–441.

Umehara, T., Nakamura, Y., Jang, M.K., Nakano, K., Tanaka, A., Ozato, K., Padmanabhan, B., and Yokoyama, S. (2010). Structural basis for acetylated histone H4 recognition by the human BRD2 bromodomain. J. Biol. Chem. *285*, 7610–7618.

Tsai, W.W., Wang, Z., Yiu, T.T., Akdemir, K.C., Xia, W., Winter, S., Tsai, C.Y., Shi, X., Schwarzer, D., Plunkett, W., et al. (2010). TRIM24 links a non-canonical histone signature to breast cancer. Nature *468*, 927–932.

Vollmuth, F., and Geyer, M. (2010). Interaction of propionylated and butyrylated histone H3 lysine marks with Brd4 bromodomains. Angew. Chem. Int. Ed. Engl. *49*, 6768–6772.

Wang, Z., Song, J., Milne, T.A., Wang, G.G., Li, H., Allis, C.D., and Patel, D.J. (2010). Pro isomerization in MLL1 PHD3-bromo cassette connects H3K4me readout to CyP33 and HDAC-mediated repression. Cell *141*, 1183–1194.

Xi, Q., Wang, Z., Zaromytidou, A.I., Zhang, X.H., Chow-Tsang, L.F., Liu, J.X., Kim, H., Barlas, A., Manova-Todorova, K., Kaartinen, V., et al. (2011). A poised chromatin platform for TGF-β access to master regulators. Cell *147*, 1511–1524.

Zeng, L., Zhang, Q., Gerona-Navarro, G., Moshkina, N., and Zhou, M.M. (2008). Structural basis of site-specific histone recognition by the bromodomains of human coactivators PCAF and CBP/p300. Structure *16*, 643–652.

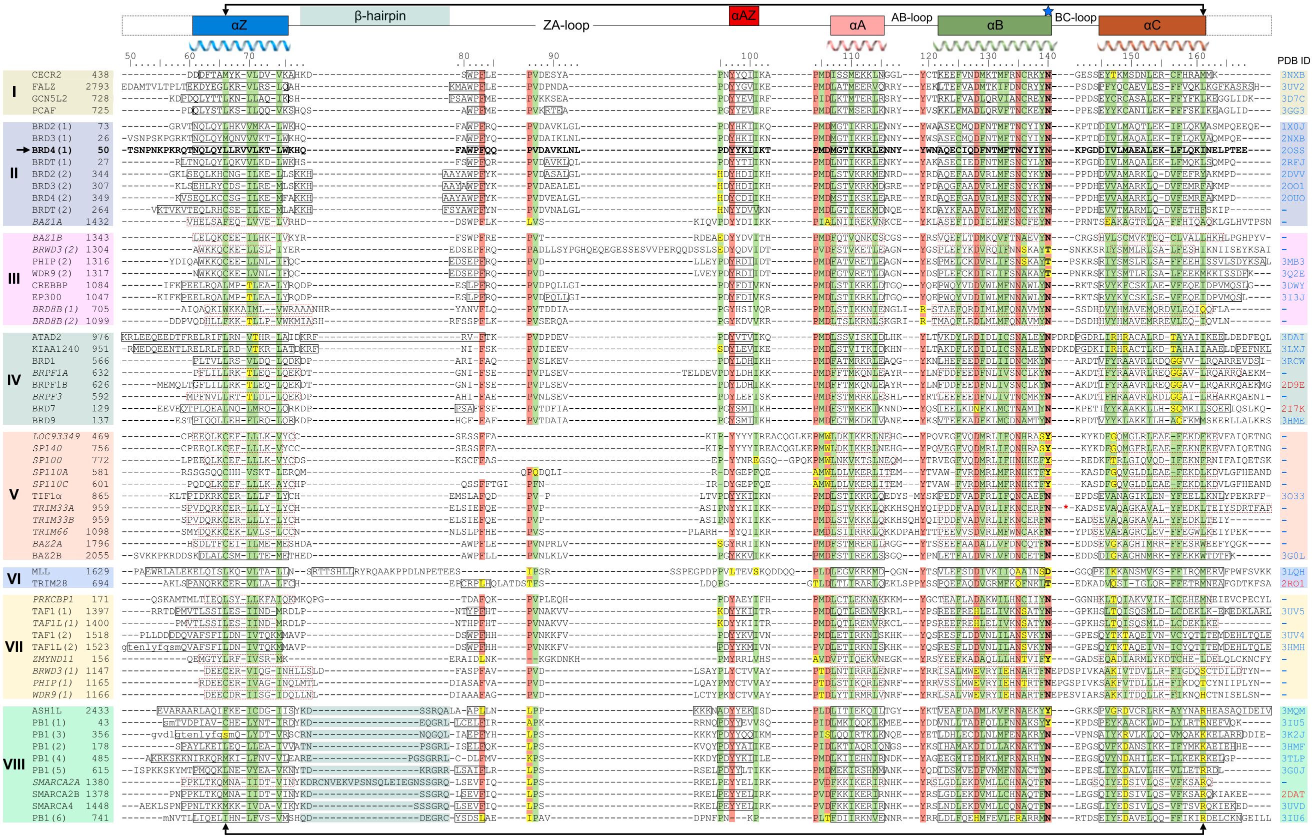

Figure S1. Structure-Guided Sequence Alignment of the Human Bromodomain Family, Related to Figure 1

Sequences are clustered by BRD families, highlighted by different colors corresponding to the eight families (Roman numbers I–VIII). The sequence region used for the generation of the phylogenetic tree is indicated by arrows. Location of bromodomain structural elements are shown and named on the top of the figure. Helices are indicated with solid black boxes (extracted from crystal structures) or red dashed boxes (predicted using PSIPRED) (Jones, 1999). Available representative structures (pdb-accession codes) are shown on the right (crystal structures in black and NMR models in red). The first bromodomain of BRD4 (bold and highlighted by an arrow) has been chosen as a reference sequence and the corresponding numbering is shown on top of the alignment. The red asterisk indicates a unique 17 residue insertion between helices B and C in the case of TRIM33A.

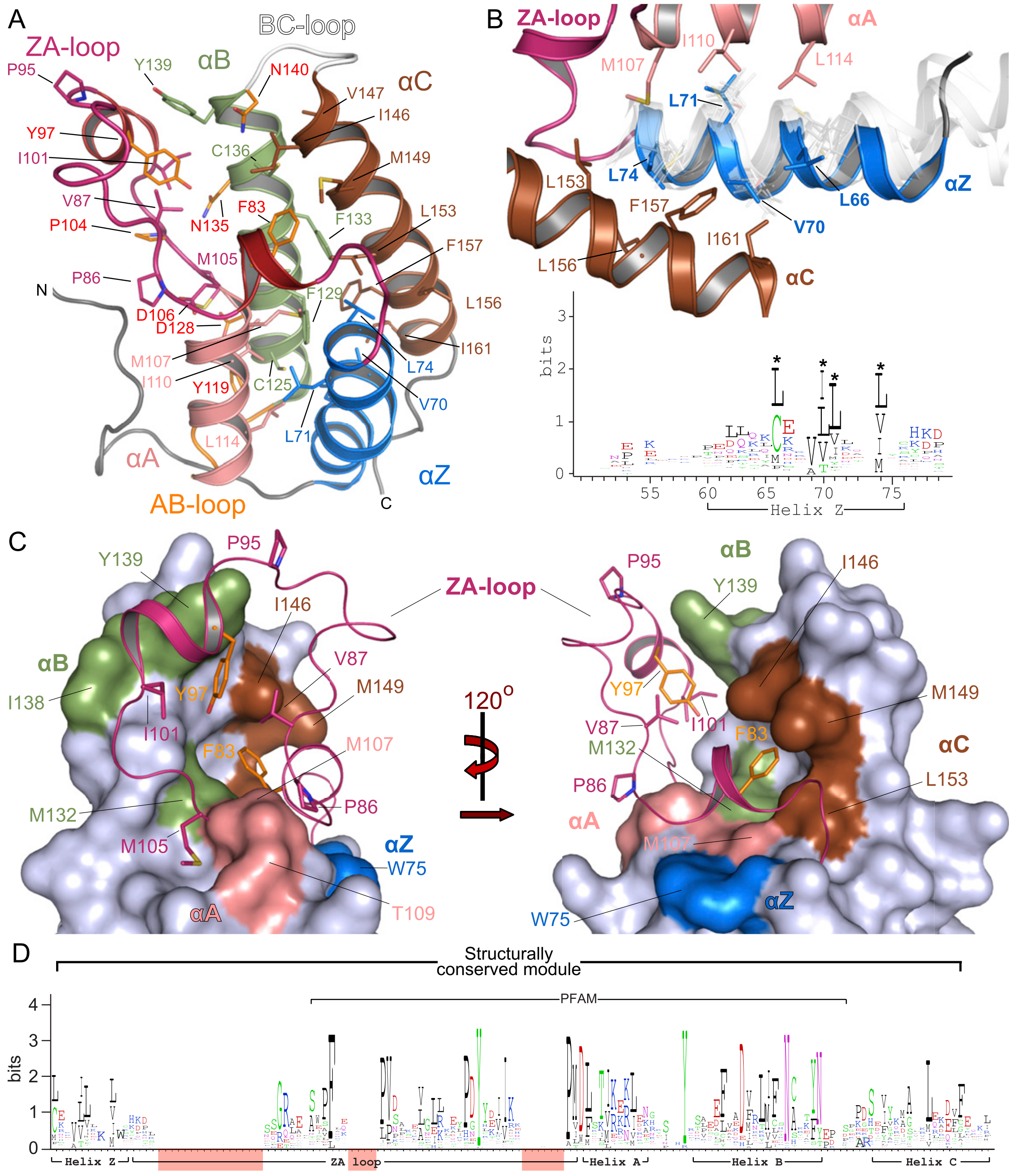

Figure S2. Structural Conservation in Helix αZ and the ZA Loop Region, Related to Figure 3

(A) Structural overview of the BRD4(1) bromodomain. Secondary structure elements and residue labels are colored as follows: α_Z blue, α_A pink, α_B green, α_C brown, ZA loop magenta.

(B) Sequence conservation in helix α_Z.

(C) Anchoring of the ZA loop to the bromodomain core. Shown are two orientations (left and right panel) of the ZA loop and its interaction with the core structure (shown as surface). The surface is colored according to the structural elements that harbor the depicted surface residues as described in panel A.

(D) Sequence conservation of for the entire family of human BRDs. Regions of low or no conservation are annotated (in pink), highlighting the advantage of employing structural and structure prediction data to define the structurally conserved BRD module.

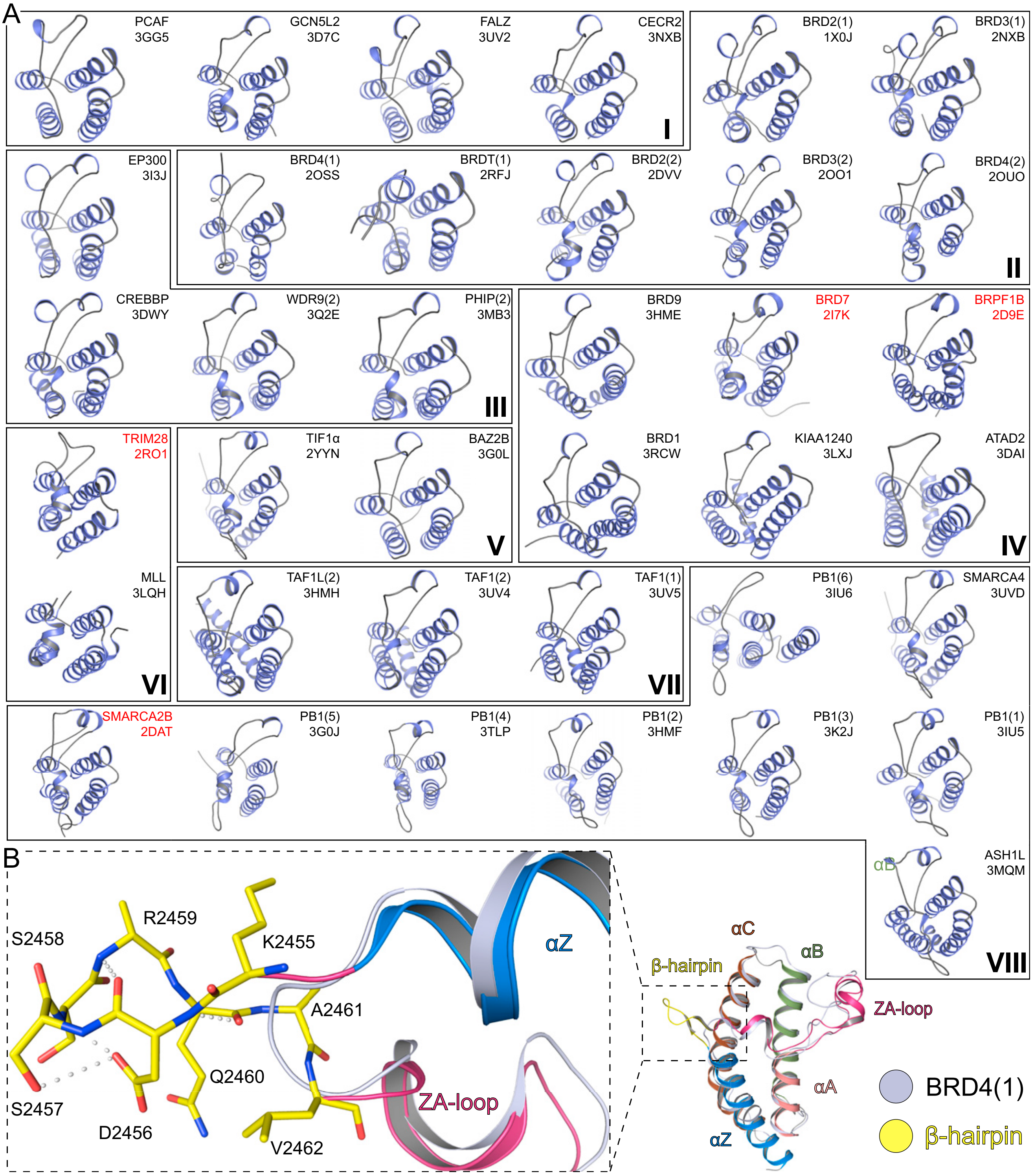

Figure S3. Overall Structure of Human Bromodomains, Related to Figure 2

(A) Ribbon diagrams of all publicly available crystal (black labels) and NMR (red labels) structures.

(B) Structural Overlay of ASH1L and the reference structure of BRD4(1). The hairpin insert, a hall mark of bromodomains of family VIII is highlighted and details are shown in the expanded view on the left panel.

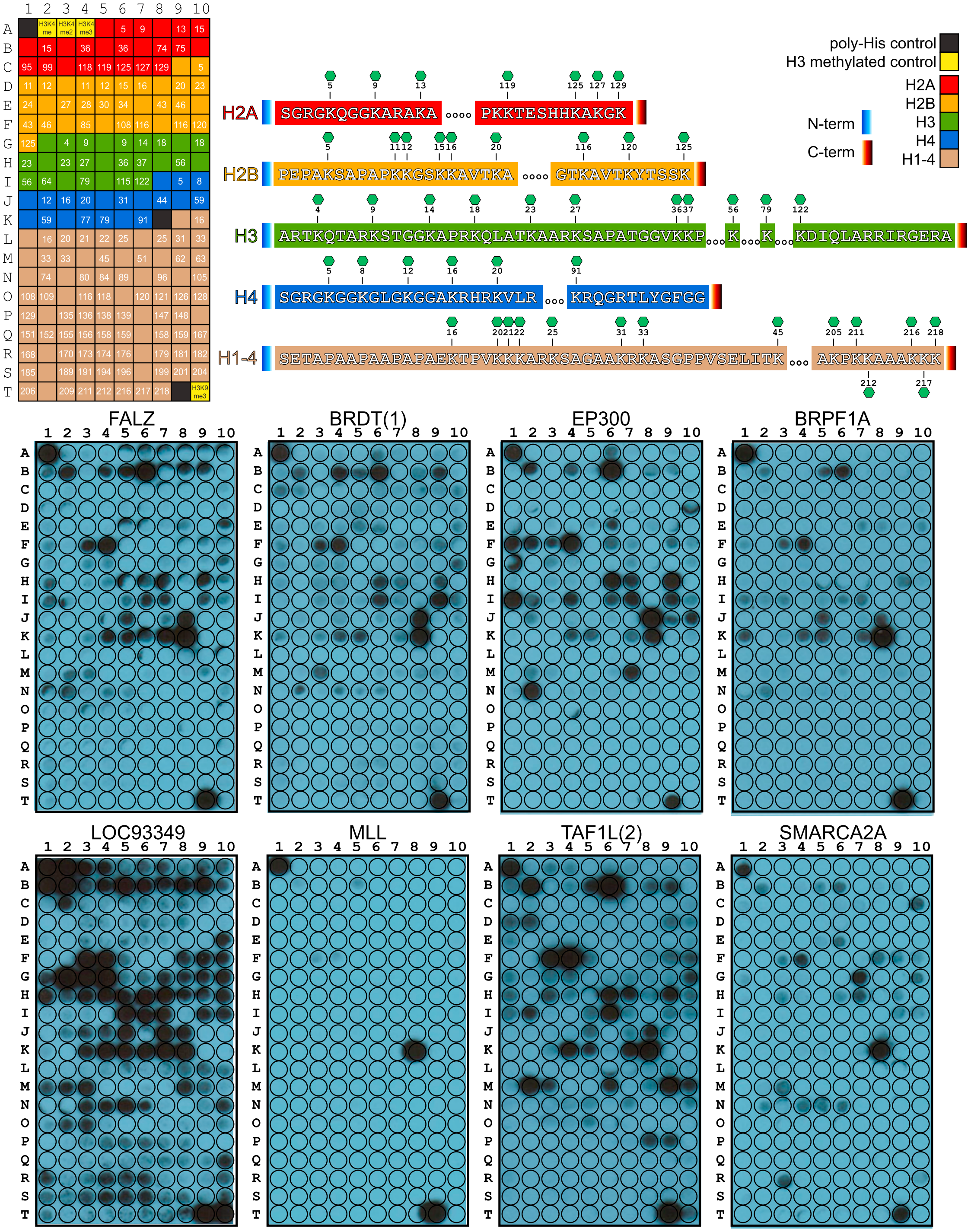

Figure S4. SPOT Membrane Layout of Figure 4 and Representative Membranes for Each Family of BRDs, Related to Figure 4

Top panel: Core (H2A, H2B, H3 and H4) and linker (H1.4) histone sequences used in the SPOT membranes of Figure 4 are shown with K_{ac} residues numbered (in the template) and highlighted (on the sequences) with a green hexagon. Boxes without a number represent control (nonacetylated) peptides for the histone marks following in the array. Lower panel: Representative membranes for each subfamily are given as follows: family I - FALZ, family II - BRDT(1), family III - EP300, family IV - BRPF1A, family V - LOC93349, family VI - MLL, family VII - TAF1L(2) and family VIII - SMARCA2A.

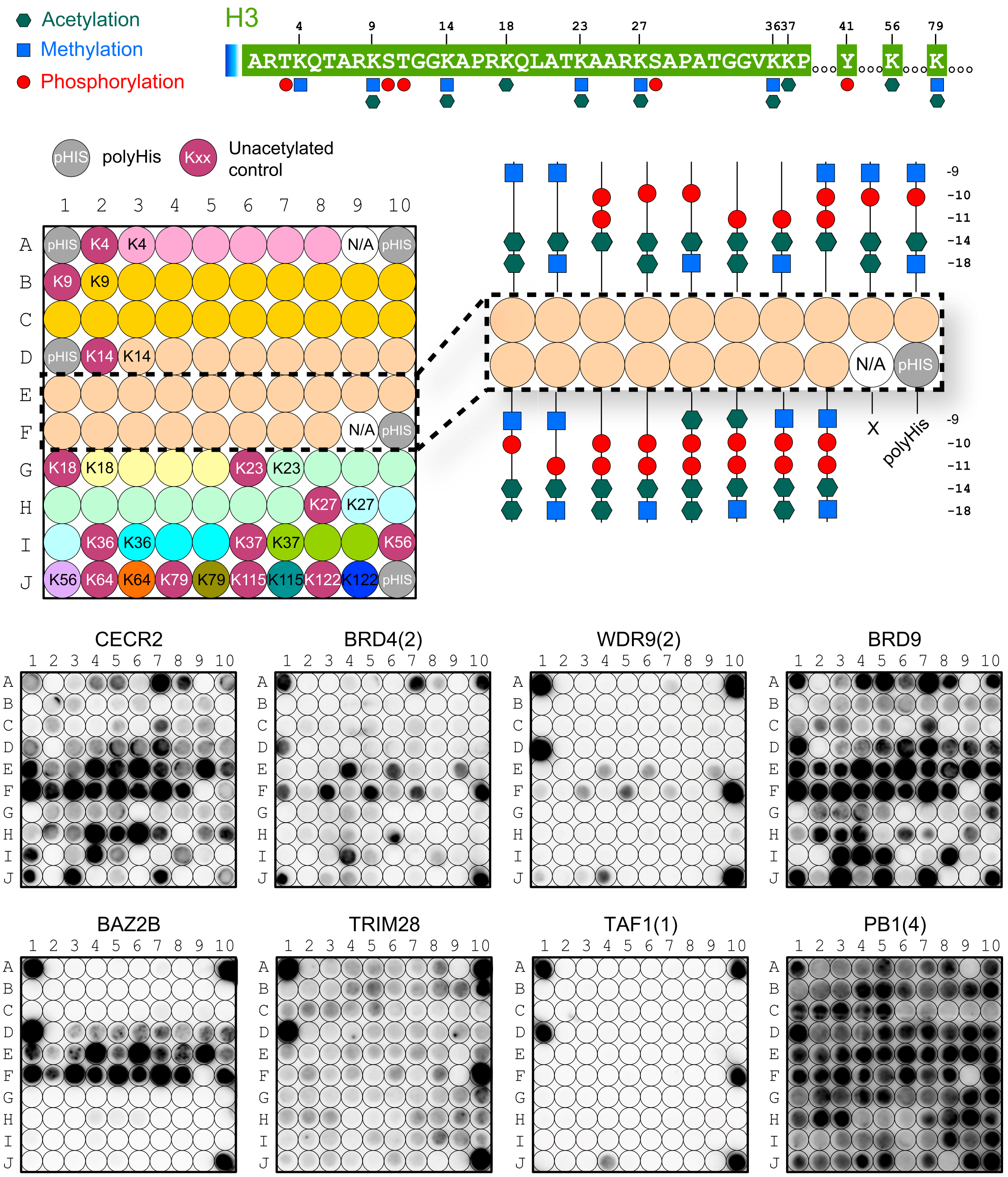

Figure S5. microSPOT Membrane Layout of Figure 5 and Representative Membranes for Each Family of BRDs, Related to Figure 5

Top Panel: The N-terminal sequence of human histone 3 is shown with highlighted marks that were studied for crosstalk, including lysine acetylation and (tri-) methylation as well as threonine and serine phosphorylation. Peptide sequences are given in the inset of Figure 5. The membrane layout highlights the position of central epitopes (numbered). The blow-up shows as an example the arrangement of marks around the H3K14$_{ac}$ epitope. Lower panel: Representative membranes for each subfamily are given as follows: family I - CECR2, family II - BRD4(2), family III - WDR9(2), family IV - BRD9, family V - BAZ2B, family VI - TRIM28, family VII - TAF1(1) and family VIII - PB1(4).

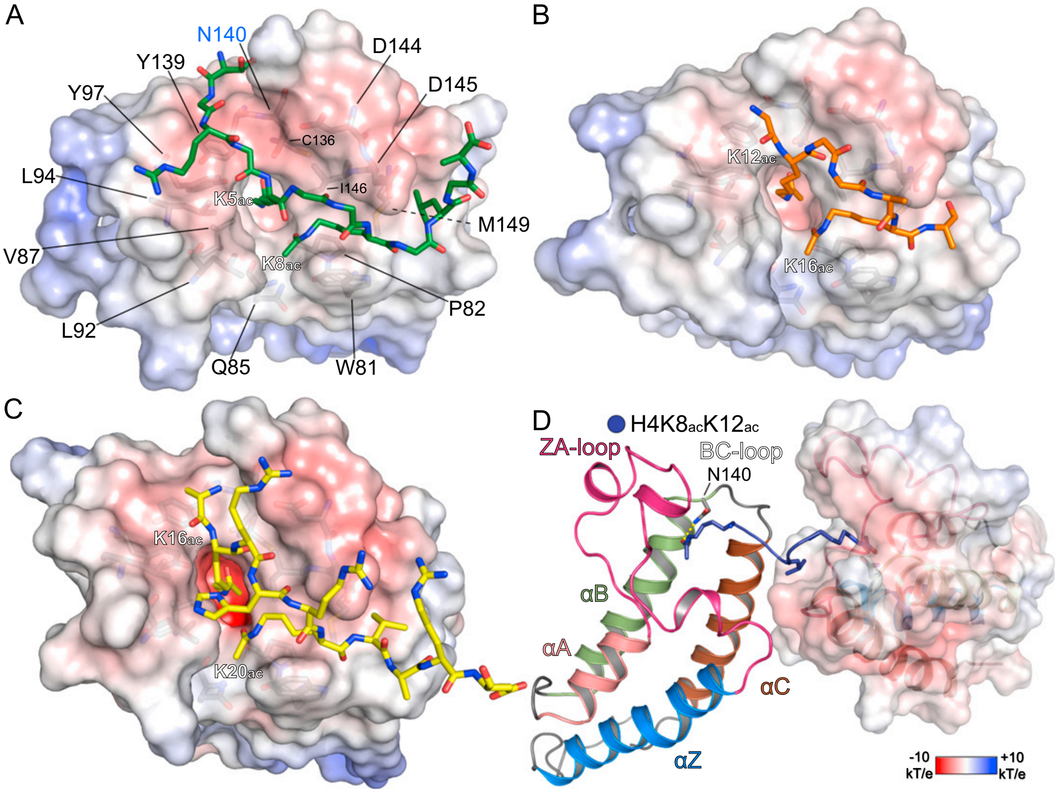

Figure S6. Binding of BRDs to Diacetylated H4 Peptides, Related to Figure 6

(A) Detail from the crystal structure of $H4_{1-11}K5_{ac}K8_{ac}$ binding to BRD4(1). The protein surface has been colored according to its electrostatic properties and key residues are annotated.

(B) Detail of $H4_{11-21}K12_{ac}K16_{ac}$ binding to the surface of BRD4(1).

(C) Detail of $H4_{15-25}K16_{ac}K20_{ac}$ binding to the surface of BRD4(1).

(D) Binding of two BRD4(1) modules to the $H4_{7-17}K8_{ac}K12_{ac}$ peptide.

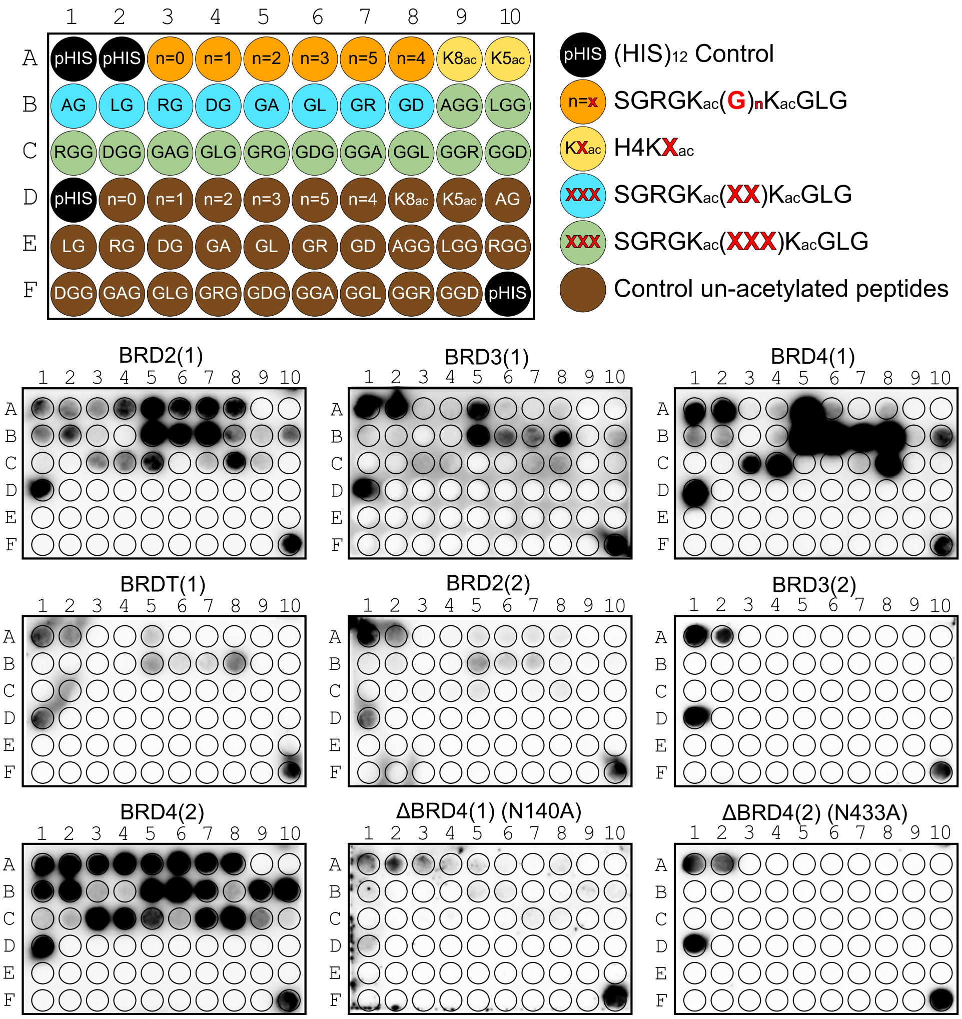

Figure S7. SPOT Membrane Layout of Figure 7 and Membranes for Each BET Bromodomain, Related to Figure 7

Membrane layout (top) of the peptides used to probe the effect on binding of a poly-glycine linker and of different residue properties between two acetyl-lysine marks on histone H4. Stained membranes of BET family bromodomains and the two inactive mutants of BRD4 are given in the lower panel.
